# A haplotype-resolved gap-free genome assembly provides novel insight into monoterpenoid diversification in *Mentha suaveolens ‘*Variegata’

**DOI:** 10.1093/hr/uhae022

**Published:** 2024-01-17

**Authors:** Hanting Yang, Can Wang, Guanru Zhou, Yuxuan Zhang, Tianxing He, Lulu Yang, Ya Wu, Zhengnan Wang, Xin Tang, Gang Chen, Zhaoyu Liu, Huanyu Tang, Hanlin Zhou, Xumei Kang, Sanyin Zhang, Liang Leng, Shilin Chen, Chi Song

**Affiliations:** Institute of Herbgenomics, Chengdu University of Traditional Chinese Medicine, Chengdu 611137, China; Pharmacy College, Chengdu University of Traditional Chinese Medicine, Chengdu 611137, China; Institute of Herbgenomics, Chengdu University of Traditional Chinese Medicine, Chengdu 611137, China; Innovative Institute of Chinese Medicine and Pharmacy, Chengdu University of Traditional Chinese Medicine, Chengdu 611137, China; Hubei University of Chinese Medicine, Wuhan 430065, China; Institute of Herbgenomics, Chengdu University of Traditional Chinese Medicine, Chengdu 611137, China; Pharmacy College, Chengdu University of Traditional Chinese Medicine, Chengdu 611137, China; Institute of Herbgenomics, Chengdu University of Traditional Chinese Medicine, Chengdu 611137, China; Pharmacy College, Chengdu University of Traditional Chinese Medicine, Chengdu 611137, China; Wuhan Benagen Technology Co., Ltd, Wuhan 430000, China; Institute of Herbgenomics, Chengdu University of Traditional Chinese Medicine, Chengdu 611137, China; Pharmacy College, Chengdu University of Traditional Chinese Medicine, Chengdu 611137, China; Institute of Chinese Materia Medica, China Academy of Chinese Medical Sciences, Beijing 100700, China; Chongqing Academy of Chinese Materia Medica, Chongqing College of Traditional Chinese Medicine, Chongqing, China; Wuhan Benagen Technology Co., Ltd, Wuhan 430000, China; Institute of Herbgenomics, Chengdu University of Traditional Chinese Medicine, Chengdu 611137, China; Wuhan Benagen Technology Co., Ltd, Wuhan 430000, China; Institute of Herbgenomics, Chengdu University of Traditional Chinese Medicine, Chengdu 611137, China; Wuhan Benagen Technology Co., Ltd, Wuhan 430000, China; Innovative Institute of Chinese Medicine and Pharmacy, Chengdu University of Traditional Chinese Medicine, Chengdu 611137, China; Institute of Herbgenomics, Chengdu University of Traditional Chinese Medicine, Chengdu 611137, China; Innovative Institute of Chinese Medicine and Pharmacy, Chengdu University of Traditional Chinese Medicine, Chengdu 611137, China; Institute of Herbgenomics, Chengdu University of Traditional Chinese Medicine, Chengdu 611137, China; Hubei University of Chinese Medicine, Wuhan 430065, China; Institute of Herbgenomics, Chengdu University of Traditional Chinese Medicine, Chengdu 611137, China; Innovative Institute of Chinese Medicine and Pharmacy, Chengdu University of Traditional Chinese Medicine, Chengdu 611137, China

## Abstract

*Mentha* is a commonly used spice worldwide, which possesses medicinal properties and fragrance. These characteristics are conferred, at least partially, by essential oils such as menthol. In this study, a gap-free assembly with a genome size of 414.3 Mb and 31,251 coding genes was obtained for *Mentha suaveolens* ‘Variegata’. Based on its high heterozygosity (1.5%), two complete haplotypic assemblies were resolved, with genome sizes of 401.9 and 405.7 Mb, respectively. The telomeres and centromeres of each haplotype were almost fully annotated. In addition, we detected a total of 41,135 structural variations. Enrichment analysis demonstrated that genes involved in terpenoid biosynthesis were affected by these structural variations. Analysis of volatile metabolites showed that *M. suaveolens* mainly produces piperitenone oxide rather than menthol. We identified three genes in the *M. suaveolens* genome which encode isopiperitenone reductase (ISPR), a key rate-limiting enzyme in menthol biosynthesis. However, the transcription levels of *ISPR* were low. Given that other terpenoid biosynthesis genes were expressed, *M. suaveolens ISPRs* may account for the accumulation of piperitenone oxide in this species. The findings of this study may provide a valuable resource for improving the detection rate and accuracy of genetic variants, thereby enhancing our understanding of their impact on gene function and expression. Moreover, our haplotype-resolved gap-free genome assembly offers novel insights into molecular marker-assisted breeding of *Mentha*.

## Introduction

The genus *Mentha*, commonly known as mint, comprises several strongly scented herb species of the Labiatae family. This herb is cultivated worldwide owing to its distinct aroma and commercial value [1]. This versatile plant contains a diverse array of components, such as essential oils and non-essential compounds, rendering it suitable for a wide range of potential applications [2]. *Mentha* essential oil has a long history of medicinal use as a digestive aid and analgesic [3]. Research showed that the *Mentha* essential oil possesses various biological activities (e.g. antioxidant, antibacterial, antiradiation, anticancer, and hypotensive) [4]. Understanding the *Mentha* genome offers valuable insights into its genetic traits and aids in identifying specific genes responsible for the aforementioned biological activities. The *Mentha* genome consists of large chromosomes and numerous small chromosomes, resulting in a wide range of chromosome numbers, typically ranging from 24 to 120. Pineapple mint (*M. suaveolens*) is the cultivated variegated form of apple mint [5]*. M. suaveolens* is a diploid (2n = 2x = 24) species that grows as a wild plant worldwide, and is widely used in the medical field owing to its numerous therapeutic properties [6].

Decoding complete genome sequence information can assist in detecting gene variation in genomes [7]. The telomere-to-telomere (T2T) genome assembly has been regarded as the ultimate goal of genome assembly [8]. However, due to the considerable diversity in chromosome numbers, obvious polyploidization phenomenon, and high heterozygosity [9], limited progress has been achieved in obtaining a high-quality genome assembly for *Mentha*. For highly heterozygous species, a haplotype-resolved genome could be combined with a T2T genome assembly to construct a superior reference genome (i.e. haplotype-resolved T2T genome) [10]. The first chromosome-level genome assemblies were generated in *Mentha longifolia* (*M. longifolia*) (2n = 2x = 24) [11]. However, a number of low-quality regions or undetectable sequence gaps in *M. longifolia* genome remained due to the technological limitations at the time. The completion of a haplotype-resolved genome assembly in kiwifruit [12], tea [13], and apple [14] provides a reference for assembling a high-quality *M. suaveolens* genome and lays the foundation for downstream analysis and precision breeding.

**Figure 1:**
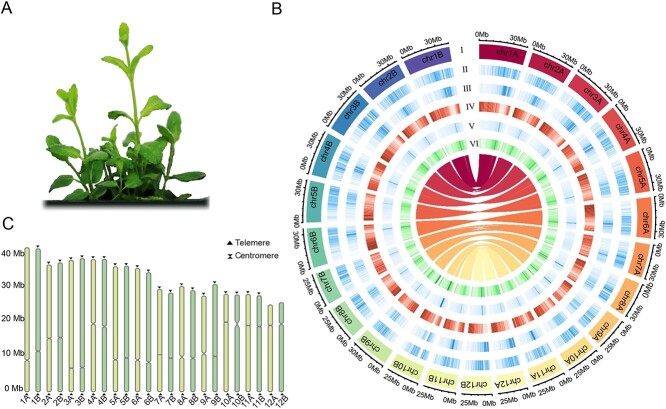
Overview of the genomic features of *M. suaveolens*. **a** Image of *M. suaveolens*. **b** Circos plot of *M. suaveolens* haplotype-resolved gap-free genomic features. I: Chromosome length. II: LTR/Copia coverage. III: LTR/Gypsy elements. IV: Gene density. V: Repeat sequence density. VI: GC content. The innermost part of the plot represents the collinear relationship between the *M. suaveolens* haplotype-resolved genomes. **c** Location of the predicted centromere region and identified telomere sequences in *M. suaveolens.* Abbreviations: GC, guanine-cytosine; LTR, long terminal repeat; Mlon, *Mentha longifolia*; Msua, *Mentha suaveolens*.

High-quality genome assembly facilitated the identification of genes encoding key enzymes in the secondary metabolite synthesis pathway [15]. Additionally, the haplotype-resolved genome is valuable in revealing the impact of genomic variation on gene function and expression [16], thus further helping us to understand the regulatory mechanisms of secondary metabolite biosynthesis [17]. Numerous recent studies focused on the biosynthesis of monoterpene compounds in *Mentha* [18]. Essential oils in *Mentha* are mainly monoterpenes, such as menthol, carvol, and pulegone. Menthol is the most abundant compound in the essential oils of most *Mentha* plants (e.g. *Mentha piperita* [19], *Mentha arvensis* [20], and *Mentha canadensis* [21])*.* However, a large number of studies have shown that piperitenone oxide (PO), rather than menthol, is the main volatile chemical component of *M. suaveolens* [22]. PO and menthol share the steps of isopiperitenone biosynthesis. Subsequently, isopiperitenone is converted by unknown reactions to produce PO rather than being converted by isopiperitenone reductase (*ISPR*) to produce menthol [11]. *ISPR* may be the key factor inducing diversity in essential oils.

In this study, we assembled the first haplotype-resolved and gap-free genome of *M. suaveolens* by integrating data from the MGI sequencing platform, Oxford Nanopore Technologies (ONT) ultralong reads, PacBio High-Fidelity (HiFi) reads, and Hi-C sequencing technologies. We further analyzed the features and mechanisms of structural variations (SVs). We also investigated the expression of terpenoid biosynthesis genes to decipher the genetic aspects that influence the accumulation of volatile terpenoids in *Mentha*. This study lays a foundation for *Mentha* genomics and provides a scientific basis for subsequent germplasm innovation and fine variety selection.

## Results

### Gap-free reference genome assembly and phased diploid genome

The *K*-mer analysis showed that the estimated genome size of *M. suaveolens* was 420.48 Mb, accompanied by a high level of heterozygosity (1.50%) and repetitive sequences (53.83%) (Fig. 1a, Fig. S1). We incorporated different sequencing platforms to develop a gap-free genome assembly for *M. suaveolens* using 29.73 Gb ONT ultralong sequencing data with N50 being 54.51 kb; 31.27 Gb HiFi sequencing data using the PacBio sequel II platform; and 71.2 Gb Hi-C sequencing data (Table S1). The matrix generated from the Hi-C short reading library based on the relationship between contig interaction intensity and position showed that 12 chromosomes were reasonably assembled (Fig. S2). The final genome size was 414.3 Mb with a contig N50 of 32.9 Mb, containing 12 chromosomes with 12 contigs (Fig. S3). The final genome size was in accordance with the estimated genome size according to the *K-*mer analysis. Based on HiFi reads, we generated two fully resolved haplotypes (termed hapA and hapB) using Hi-C and ONT ultralong reads for assisted haplotyping (Fig. 1b). The assembled haplotypes contained 12 pseudomolecules with a total length of 401.9 and 405.7 Mb, respectively. Moreover, the contig N50 of hapA was 35.1 Mb, while that of hapB was 36.3 Mb (Table 1). The final genome size was similar to that estimated by the *K*-mer analysis. Finally, according to the characteristic repetitive base sequence of the telomere region (CCCTAAA/TTTAGGG), we identified 45 telomeres and 24 centromeres in 24 pseudochromosomes of the haplotype-resolved gap-free genome of *M. suaveolens*; three telomeres remain to be determined (Fig. 1c, Table S2). Compared with the 3266 gaps that remained in the *M. longifolia* genome, the absence of gaps in all chromosomes represented the continuity of the *M. suaveolens* genome assembly (Fig. S4).

**Table 1:** Summary statistics of *M. suaveolens* genome assembly

**Feature**	***M. longifolia* V3** [11]	**Gap-free genome (*M. suaveolens*)**	**HapA (*M. suaveolens*)**	**HapB (*M. suaveolens*)**
Assembly total length (Mb)	469.1	414.9	401.9	405.7
Protein-coding genes	42 107	31 251	31 688	32 011
Contig number	3586	12	12	12
Contig N50 (bp)	394 381	36 297 221	35 112 294	36 357 466
Number of gaps in chromosomes	3266	0	0	0
Number of telomeres	0	22	23	22
Number of definite centromeres	0	12	12	12
Genome BUSCOs (%)	91.5	99.2	99.1	99.1

Using the hapA genome as a reference, >99.1% of the core conserved genes (i.e. 1600 of 1614 Benchmarking Universal Single-Copy Orthologs (BUSCOs) were completely assembled (Table 1). We mapped the reads to evaluate genome consistency, resulting in 97.17% mapping rate and 99.96% coverage. The assembly accuracy (estimated as qualification value) was 46.46, indicating the construction of the haplotype-resolved and gap-free genome assembly of *M. suaveolens* with high integrity and accuracy (Table S3). Through this process, we completed the first high-quality haplotype-resolved gap-free genome assembly for *Mentha*.

Repetitive sequences accounted for 61.8% of the *M. suaveolens* genome (total length: 249,298,078 bp), which were mainly composed of tandem repeats and dispersed repeats. Long terminal repeats (LTRs) accounted for 34.08%, including Gypsy (19.7%) and Copia (10.02%), with a total length of 137,470,926 bp (Table S4). We further assessed genome completeness by LTR Assembly Index (LAI) LAI evaluation and, obtained 20.29 LAI value. We predicted 31,688 coding genes in the genome, with an average messenger RNA length of 3957 bp, an average coding sequence length of 1164 bp, and an average number of 5.12 exons per gene (Table S5, S6). Approximately 99% of the core conserved genes (1598 of 1614 BUSCOs) were complete in the *M. suaveolens* genome annotation (Table S7). Functional annotation of genes was performed by tagging gene functions and associated metabolic pathways involved based on various databases, including predictions of motifs, domains, protein functions, and the metabolic pathways in which they were involved. The results showed that 92.74% (29,388) of the genes were annotated in at least one database (Table S8). Moreover, we identified a total of 162 microRNAs (miRNAs), 534 transfer RNAs (tRNAs), 333 ribosomal RNAs (rRNAs), and 2135 small nuclear RNAs (snRNAs) (Table S9).

### Phylogenetic and whole genome duplication analysis

We sought to explore the phylogenetic position of *M. suaveolens* through comparative genomic analysis. Therefore, we downloaded the protein sequences of 14 species (i.e. *Vitis vinifera*, *Artemisia carvifolia*, *Catharanthus roseus*, *Lycopersicon esculentum*, *Sesamum indicum*, *Scutellaria baicalensis*, *Schizonepeta tenuifolia*, *Thymus quinquecostatus*, *M. longifolia*, *Salvia splendens*, *Salvia miltiorrhiza*, *Arabidopsis thaliana*, and *Oryza sativa*). The results revealed a total of 56,244 homologous gene families comprising 457,838 genes across all species; of note, 5810 gene families were shared by all species. The *M. suaveolens* genome had 1334 unique gene families with 1609 genes (Fig. S5; Table S10). The Kyoto Encyclopedia of Genes and Genomes (KEGG) analysis revealed that a significant proportion of the unique gene families were mainly enriched in the spliceosome and phenylpropanoid biosynthesis (Fig. S6). We further focused on the Labiatae species, which are representative of essential oil-containing plants (i.e. *M. suaveolens*, *M. longifolia*, *S. tenuifolia*, and *T. quinquecostatus*). There were 1422 unique gene families in *M. suaveolens* genome, while 8564 genes families were shared by all four species (Fig. S7). The shared gene families were mainly enriched in endocytosis and glycolysis, and participated in monoterpenoid and terpenoid backbone biosynthesis (Fig. S8). The unique gene families of *M. suaveolens* were mainly enriched in spliceosome and phenylpropanoid biosynthesis (Fig. S9).

**Figure 2:**
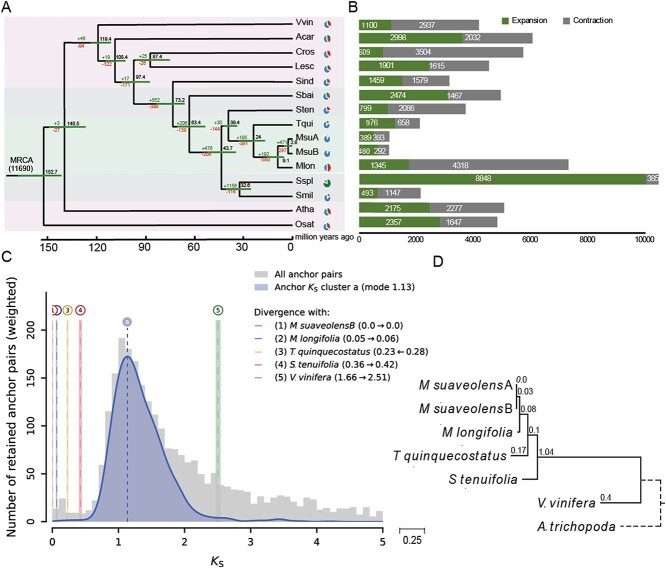
Phylogenetic analysis and identification of WGD events. **a** The phylogenetic tree constructed based on 178 single-copy genes*.***b** The numbers of expanded or contracted gene families among 14 species. **c** WGD signatures in Labiatae (Rate-adjusted mixed *K*s distribution for *M. suaveolens*). Gray: the anchor pair *K*s distribution of this *M. suaveolens*, the vertical dashed lines labeled ‘a’ indicated WGD age estimates based on *K*s. **d** Phylogram of *M. suaveolens*, *Mentha longifolia*, *Schizonepeta tenuifolia*, *Thymus quinquecostatus*, and *Vitis vinifera* by *K*s rates, with branch lengths. Abbreviations: Acar, *Artemisia carvifolia*; Atha, *Arabidopsis thaliana*; Cros, *Catharanthus roseus*; *K*s, synonymous substitutions per synonymous site; Lesc, *Lycopersicon esculentum*; Mlon, *M. longifolia*; *M. suaveolens*, *Mentha suaveolens*; Osat, *Oryza sativa*; Sbai, *Scutellaria baicalensis*; Sind, *Sesamum indicum*; Smil, *Salvia miltiorrhiza*; Sspl, *Salvia splendens*; Sten, *Schizonepeta tenuifolia*; Tqui, *Thymus quinquecostatus*; Vvin, *V. vinifera*; WGD, whole genome duplication.

By utilizing >100 single-copy orthologous genes of *M. suaveolens* and the 14 species to construct a phylogenetic tree, we estimated that the divergence time between Labiatae species and *Sesamum indicium* (a Pedaliaceae species) was 73.2 million years ago (Mya). The estimated divergence time between *Mentha* and *T. quinquecostatus*, and between *M. suaveolens* and *M. longifolia* was 24 and 9.1 Mya, respectively. (Fig. 2a).

Furthermore, we identified 383 expanded and 393 contracted gene families in the *M. suaveolens* genome (Fig. 2b). The KEGG analysis showed that the contracted gene families mainly participated in the mitogen-activated protein kinase (MAPK) signaling pathway and phenylpropanoid biosynthesis, while the expanded gene families were mainly enriched in ubiquitin-mediated proteolysis, phenylalanine metabolism, and terpenoid backbone biosynthesis pathways (Fig. S10). Gene ontology (GO) analysis demonstrated that the contracting gene families were mainly enriched in polysaccharide binding and the apoplast, as well as in the terpenoid biosynthetic process; the expanded gene families were mainly enriched in FDA binding (Fig. S11).

The number of synonymous substitutions per synonymous site (*K*s) analysis depicted that *M. suaveolens* had experienced a whole genome duplication (WGD) event at *K*s ~ 1.13, which was shared with Labiatae (Fig. 2c). The *K*s plot for the paralogous genes did not show any signs of *M. suaveolens*-specific WGD. In addition, the dot-plot analysis between *M. suaveolens* with *M. Longifolia*, *T. quinquecostatus* and *S. tenuifolia* showed a 1:1 pattern, which was further confirmed *M. suaveolens* had experienced a WGD event (Fig. S12). By constructing a phylogram of *M. suaveolens*, *M. longifolia*, *S. tenuifolia*, and *T. quinquecostatus* using the *K*s rates, we were able to put the estimated values of the same event more closely together. Moreover, correct phylogenetic localization of WGD was achieved in this lineage (Fig. 2d).

### SV in *M. suaveolens* may influence terpenoid biosynthesis

**Figure 3:**
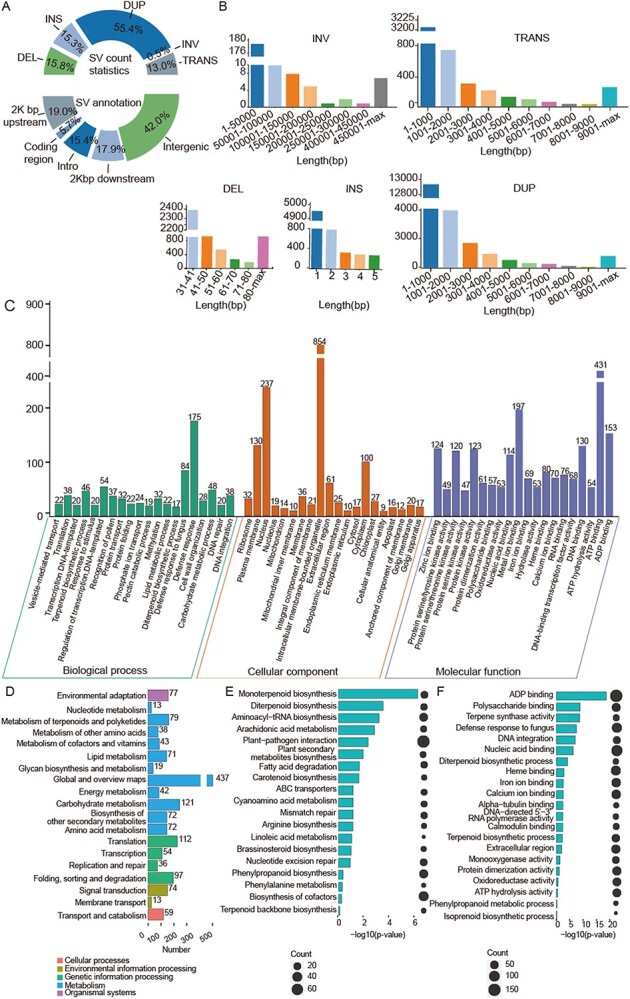
Analysis of SVs and related genes. **a** Quantity statistics and annotation of SV. **b** SV length statistics for INV, DEL, TRANS, INS, and DUP. **c** GO annotation of SV-affected genes. **d** KEGG annotation statistics of SV-affected genes. **e** KEGG enrichment analysis of SV-affected genes. **f** GO enrichment analysis of SV-affected genes. Abbreviations: DEL, deletion; DUP, duplication; GO, Gene Ontology; INS, insertion; INV, inversion; KEGG, Kyoto Encyclopedia of Genes and Genomes; SV, structural variation; TRANS, translocation.

The resolution of two complete haplotypes enabled a genome variation analysis using Synteny and Rearrangement Identifier (SyRI) (Fig. 3a). A total of 3,508,508 variants were identified, including single nucleotide polymorphisms, indels, and SVs (Table S11). A total of 41,135 SVs were discovered, including 6519 deletions (15.8%) (length distribution: 31–40 bp); 6277 insertions (15.3%) ranging from 1 to 5 bp (with 1-bp insertions being the most abundant type); 22,774 duplications (55.4%) and 5352 translocations (13%) (with lengths generally <1000 bp); and 213 inversion (0.5%) (with lengths generally <5000 bp) (Fig. 3a, b). Large SVs, such as duplications and inversions, were observed in the *M. suaveolens* genome (Fig. S13). Overlapping of SVs and gene annotation revealed that 8874 SVs were located within 2 kb upstream of genes (19.0%), 2653 SVs were located within the coding region of genes (5.7%), 7212 SVs were located within introns (15.4%), 8349 SVs were located within 2 kb downstream of genes (17.9%), and 19 605 SVs were located within intergenic regions (42%).

GO and KEGG annotations, followed by enrichment analysis of SV-affected genes, were performed to link functions and metabolic pathways with these genes. The results of the GO analysis showed that a large proportion of genes affected by SVs were involved in the defense response and terpenoid biosynthesis pathway (Fig. 3c). According to the KEGG analysis, numerous genes affected by SVs were involved in the global and overview maps, as well as the metabolism of terpenoids and polyketides (Fig. 3d). Based on the GO and KEGG enrichment analyses, several terpene biosynthesis-related terms were identified among the top enriched groups. For example, GO terms monoterpenoid biosynthesis and diterpenoid biosynthesis, and KEGG term terpene synthase activity ranked first, second, and third respectively. These results implied that SV plays important roles in the synthesis of aromatic compounds and production of volatile terpenoids (Fig. 3e, f).

### Piperitenone oxide is the main volatile compound in *M. suaveolens*

**Figure 4:**
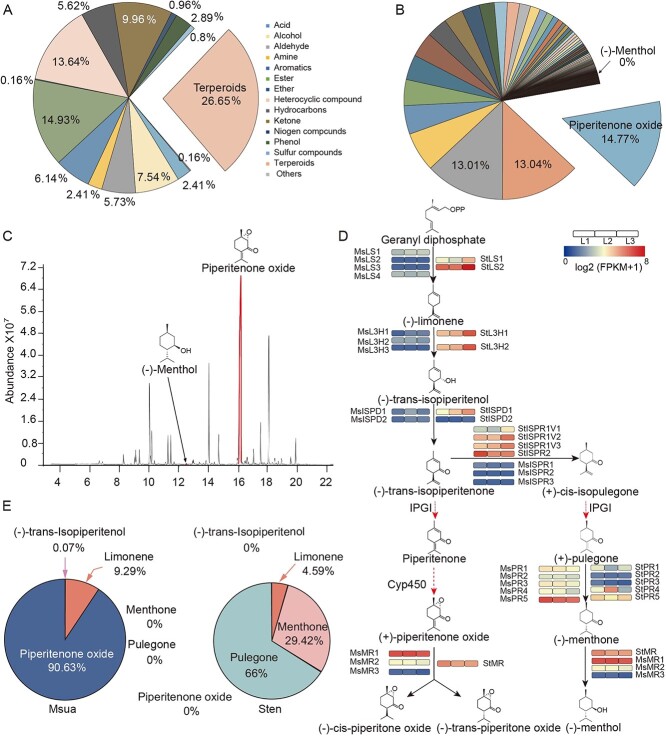
Analysis of volatile metabolites in *M. suaveolens.***a** Classification of all volatile metabolites. **b** Classification of terpenoid compounds in *M. suaveolens.***c** The position of menthol and piperitenone oxide peaks in the total ion flow diagram (TIC diagram) of the QC sample mass spectrum. **d** The main biosynthetic pathway of monoterpene biosynthesis in *Mentha*. The dotted line shows the unidentified enzyme. The expression (log_2_(FPKM+1)) of each gene is shown as a heatmap in leaves (L); 1, 2, and 3 represent three biological replicates. **e** Relative proportion of five metabolites in the proposed monoterpene biosynthetic pathway. Abbreviations: FPKM, fragments per kilobase of exon model per million mapped fragments; IPGI, isopulegone isomerase; ISPD, isopiperitenone dehydrogenase; ISPR, isopiperitenone reductase; L3H, limonene-3-hydroxylase; LS, 4-hydroxy-3-methylbut2-en-1-yl diphosphate reductase; *M. suaveolens*/Msua, *Mentha suaveolens*; PR, pulegone reductase; Sten, *Schizonepeta tenuifolia*; TIC, total ion chromatogram; QC, quality control.

The analysis of volatile metabolites revealed that terpenoids accounted for the largest proportion (26.65%) (Fig. 4a). The downstream product of isopiperitenone, PO, was predominant among all terpenoid compounds (14.77%) (Fig. 4b). The mass spectra of major volatile metabolites are shown in Fig. 4c. Subsequently, samples obtained from different stages of development were analyzed. The results showed that the primary components of *M. suaveolens* volatile oils were consistent with previous findings. Furthermore, menthol was almost undetectable during the growth and development process (Fig. S14). This discovery highlights the need for further exploration of the metabolic pathways and products of *M. suaveolens* to gain a more comprehensive understanding of its growth and medicinal value.

### Monoterpene biosynthesis pathway in *Mentha*

Previous research showed that *Mentha* plants use the methylerythritol phosphate pathway as the main source of C5 raw material for essential oil production and other monoterpene biosynthesis [23, 24]. Geranyl diphosphate produces menthol and pulegone via two pathways catalyzed by a series of enzymes [25] (Fig. 4d). We identified genes that may participate in monoterpene biosynthesis through Basic Local Alignment Search Tool (BLAST) search of orthologous genes with known functions in the *M. suaveolens* genomes (Table S12). Regarding genes involved in monoterpenoid biosynthesis (Table S13), four limonene synthase (LS) genes, three limonene-3-hydroxylase (L3H) genes, two isopiperitenone dehydrogenase (ISPD) genes, three ISPR genes, three menthol reductase (MR) genes, and five pulegone reductase (PR) genes exhibiting high homology with previously reported genes were identified in the *M. suaveolens* genomes (Fig. S15, S16).

We analyzed the monoterpene biosynthesis pathway in *Mentha* by combining transcriptome and metabolome data. After trans-isopiperitenone, the *Mentha* monoterpene biosynthetic pathway diverges into two branches; one branch leads to the formation of PO, while the other leads to the formation of menthol. The first step of the menthol biosynthetic branch, i.e. from isopiperitenone to isopulegone, is catalyzed by *ISPR*. Thus, the expression and function of *ISPR* in *M. suaveolens* may be the main reason responsible for the competitional formation of PO over menthol. A possible explanation for the lack of menthol biosynthesis in *M. suaveolens* is the loss of *ISPR* genes in the *M. suaveolens* genome. Nevertheless, three *ISPR* genes were identified, while their expression was consistently limited, e.g. *MsISPR2,* and *MsISPR3*. The fragments per kilobase of exon model per million mapped fragments (FPKM) values were zero in all tested tissues (leaf, root, stem) and growth stages (Fig. S17). However, the FPKM values of three *MR* genes encoding the enzyme catalyzing two reactions in downstream branches, were relatively high. Consequently, we hypothesized that these *MRs* participate in the PO biosynthetic branch. In *S. tenuifolia*, pulegone and menthone were identified as the intermediates of menthol biosynthesis, accounting for the largest proportions of monoterpenes (Fig. 4e). Two *ISPR*s were identified in the *S. tenuifolia* genome, and had high FPKM values compared with three *ISPRs* detected in the *M. suaveolens* genome.

**Figure 5:**
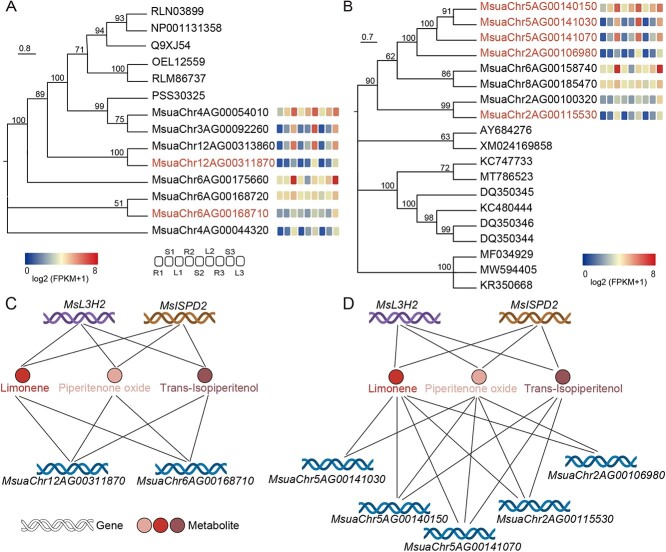
Search for candidate piperitenone oxide biosynthesis genes. The phylogenetic relationship of the *M. suaveolens* hapA genome. *NTF2* (**a**) or *CYP71D* (**c**) and their orthologous genes were constructed. The expression (log_2_(FPKM+1)) of each gene is shown as a heatmap. L: leaf, R: root, S: stem. Correlation of candidate *NTF2* (**b**) or *CYP71D* (**d**) genes with the intermediate genes and metabolites of the monoterpene biosynthesis pathway in *M. suaveolens*. A *CYP71D* network was constructed with *r* > 0.8, *p* < 0.05, and FPKM >1. A *NTF* network was constructed with *r* > 0.6, *p* < 0.05, and FPKM >1. Abbreviations: FPKM, fragments per kilobase of exon model per million mapped fragments.

### Putative enzyme catalyzing isopiperitenone into PO

At least two enzymes were required to produce PO from isopiperitenone, namely an unknown isopulegone isomerase (IPGI) and a terpene epoxidase. Isopiperitenone is converted to piperonone via a reaction catalyzed by *IPGI*, and piperitenone subsequently produces PO via a reaction catalyzed by terpene epoxidase. It was reported that Δ5-3-ketosteroid isomerase, a member of the nuclear transport factor 2 (NTF2) family [26], from *Pseudomonas putida* possesses *IPGI* activity [27]. In addition, *CYP71D* is a type of terpene epoxidase that catalyzes the conversion of piperonone to PO [28, 29]. The key enzymes in *Mentha* remain to be determined. Eight *NTF2* and eight *CYP71D* genes were found in the *M. suaveolens* genome using homologous sequence alignment and co-expression analysis (Fig. 5a, b). The expression of two established monoterpene biosynthesis genes (i.e. *L3H2* and *ISPD2*) was positively correlated with the accumulation of PO and compounds in its biosynthesis pathway (i.e. limonene, and trans-isopiperitenol). The highest expression and greatest accumulation of these gene and compounds were detected in the leaf, followed by the stem and root. According to the co-expression analysis of eight *NTF2* and eight *CYP71D* with two known genes (*L3H2* and *ISPD2*) and metabolites (PO, limonene, trans-isopiperidol) involved in monoterpene biosynthesis, two *NTF2* genes (MsuaChr6AG00168710 and MsuaChr12AG00311870) and five *CYP71D* genes (MsuaChr5AG00141030, MsuaChr5AG00140150, MsuaChr5AG00141070, MsuaChr2AG00115530, MsuaChr2AG00106980) exhibited strong positive correlations, and were identified as candidates genes (Fig. 5c, d). These genes may participate in the biosynthesis of monoterpenes or related precursors and play a crucial role in converting isopiperitenone into PO.

## Discussion

*Mentha* has been cultivated for >400 years in China, and breeding of *Mentha* has been highly valued [30]. A high-quality genome plays an important role in explaining the origin, evolution, and spread of plants, effectively protecting germplasm resources [31] and assisting in the exploration of new genes and efficient germplasm innovation [32]. For *Mentha*, two genome versions of *M. longifolia* have been released and updated since 2017 [11, 33]. High-quality genomes of other *Mentha* species have not been released thus far. Regarding other Labiatae species, the high-quality genome of Japanese catnip (*S. tenuifolia*) (a species closely related to *Mentha*) was recently released [34]. In addition, high-quality genome assembly was generated in *Salvia splendens* (scarlet sage) [35], *Salvia miltiorrhiza* [36], *Salvia officinalis* (sage) [37], *Perilla frutescens* [38], and *Thymus mongolicu* [39]*.* Thus far, although genomes of multiple Labiatae plants have been published, a T2T genome assembly for the family has not been established thus far. In this study, the first haplotype-resolved gap-free genome of *Mentha* was generated.

WGD events occurred occasionally in Labiatae. A very recent polyploidization occurred in *Perilla frutescens* within 10 000 years [38]. Two WGD events and a WGT event were reported in *Salvia splendens* genome at *K*s = ~0.08, ~0.2, and 0.6–0.8, respectively [35]. Two WGD events were reported in the *T. quinquecostatus* genome at *K*s = ~0.07 and ~ 1.22, respectively [39]. Two large-scale gene duplications were reported in the *S. tenuifolia* at *K*s = 0.1–0.2 and ~ 1, respectively [34]. The *K*s plot for the paralogues of *M. suaveolens* revealed that its genome was subjected to a WGD event, which was shared with Labiatae. However, the dot-plot analysis between *M. suaveolens* with *M. Longifolia*, *T. quinquecostatus*, and *S. tenuifolia* showed a 1:1 pattern (Fig. S18). The peaks observed at *K*s = ~0.07 in *T. quinquecostatus* and at *K*s = 0.1–0.2 in *S. tenuifolia* were identified as suspicious peaks. Subsequently, we extracted the corresponding collinear regions of *S. tenuifolia* and *T. quinquecostatus* at *K*s = ~0.07 (Fig. S19). The results showed that the corresponding peaks are more likely to be caused by tandem repeats, replication, and insertion of certain genomic regions, rather than a WGD event.

Several reports demonstrated the chemical diversification of *Mentha* species [9]. Gain of enzyme gene copies usually accounts for the accumulation of specific metabolites, while loss of enzyme genes may result in paucity. For example, Liao et al. found that amorpha-4,11-diene synthase (*ADS*) gene expansion was significantly related to the levels of artemisinin [40]. Wang et al. revealed that loss of 12 exons of dammarenediol synthase (*DDS*) in *Aralia elata* may lead to low levels of dammarane-type saponin in *A. elata* [41]. The lack of artemisinin production in *Artemisia argyi* may be attributed to the partial deletion of the *ADS* gene and loss of function of the ADS homologue [42]. Consequently, we speculated that *ISPR* gene loss may cause the low menthol production in *M. suaveolens*. Surprisingly, three *ISPR* copies were identified, with zero and very low FPKM values for two and one ISPR copies, respectively. In addition to differences in the expression of key enzyme genes, we observed other factors that may be involved in the intra- and inter-species chemical diversity of terpenes in *Mentha*. Wang et al. discovered that SV had a great impact on gene function (e.g. terpene compound synthase genes) [43]. The total number of genes in *M. suaveolens* is 31 688; of those, 5493 genes (17.3%) were affected by SVs. Based on the GO and KEGG enrichment analyses, we suspected that SVs impact the synthesis and metabolism of volatile terpenoids in *M. suaveolens*. For example, SVs affected 11 genes (*HDS*: 4-hydroxy-3-methylbut-2-en-1-yl diphosphate synthase, four *LS*, three *ISPR*, and three *MR*) in the monoterpenoid pathway of *M. suaveolens* (Table S14). These variations can possibly affect the chemical composition and biological activity of plants. These findings provide valuable insights into the genetic basis of terpenoid biosynthesis and highlight the potential regulatory effects of SVs on this important pathway.

In conclusion, this work presents the first haplotype-resolved and gap-free genome of *Mentha*, thereby providing a solid scientific basis for investigating its origins. Additionally, it provides valuable resources for precise gene annotation and functional studies in the future. Using the chemical diversity of *Mentha* as the starting point, we explored the impact of SVs on the synthesis of terpenoid compounds, which ultimately affects the mint aroma characteristics. In addition, this study putatively identified two previously unknown key enzymes in the biosynthetic pathway of *Mentha* monoterpenes. Thus, this research may provide a ‘gold standard’ reference genome of *Mentha* and facilitate molecular marker-assisted breeding.

## Materials and methods

### Plant materials and sequencing

Diploid (2n = 2x = 24) *M. suaveolens* plants were obtained from the Wangcheng District, Changsha City, Hunan Province, China. Fresh leaves of *M. suaveolens* were collected for ONT ultralong, PacBio HiFi, and Hi-C sequencing. The MGI DNBSEQ-T7 platform was used to generate next-generation sequencing data. The ONT PromethION sequencer was used to generate ONT ultralong reads. A PCR-free SMRTBell library was constructed using high-quality purified long reading DNA for PacBio HiFi sequencing. Hi-C libraries were constructed and sequenced using BGI platform. Stems, leaves, and roots of *M. suaveolens* were frozen in liquid nitrogen and stored at −80°C for transcriptome and metabolome analyses.

### Genome assembly and pseudochromosome construction

The genome size and heterozygosity of *M. suaveolens* were estimated using K-mer analysis (K = 19) [44]. ONT ultralong sequencing data were assembled using nextDenovo (option: read_cutoff =1 k, blocksize = 1 g, nextgraph_options = −a 1; https://github.com/Nextomics/NextDenovo), Canu, and Flye (option: -I 3 -m 10 000). The PacBio HiFi data were assembled using Hifiasm [45]. Hi-C interaction is used to determine the strength of associations between different contigs in valid data and cluster contigs. Contigs were clustered, ordered, and oriented to chromosomes using ALLHiC [46]. Next, the ordered and oriented contigs were manually sequenced and oriented with the Juicebox software [47]. The haplotype-resolved, chromosome-level genome was obtained, remained six gaps. The corrected ONT and PacBio HiFi reads were used to align the chromosome-level genome using Winnowmap (v1.11, option: k = 15, –MD) [48]. The sequence with the best alignment was used to replace the corresponding sequence in the gap region. Finally, we obtained the haplotype-resolved gap-free genome of *M. suaveolens*. We use LTR Finder and LTR Retriever calculates LAI to evaluate repeat elements [49].

### Identification of telomeres and prediction of centromere regions

The sequence of CCCTAAA was used to identify telomeres by medaka_Consu (option: -m r941_min_high_g360; https://github.com/nanoporetech/medaka). The centromeres of *M. suaveolens* were predicted as previously reported [10]. We calculated the coverage of Tandem Repeats Finder (TRF; short tandem repeat sequences) and gene coverage in the filamentous region, with 100 k as the window size using bedtools [50]. The continuous high TRF coverage and very low gene coverage of each chromosome could be used to predict the centromere position.

### Genome annotation

We predicted the LTR sequences using RepeatModeler (option: BuildDatabase -name mydb; RepeatModeler -database mydb -pa 10) and LTR_FINDER (option: -threads 16 -harvest_out -size 1 000 000 -time 300) software. Redundant LTR sequences were removed using LTR_ retriever (option: - threads 16) [51]. The repeat sequences were masked using RepeatProteinMask (option: - noLowSimple - pvalue 0.0001). The gene structures of *M. suaveolens* were predicted combined with integrating *ab initio* gene prediction, homologous proteins, and transcriptome annotation. Functional annotation of genes was identified based on sequence and motif similarity. The tRNA sequences in the genome were identified by tRNAscan SE software [52]. The rRNA and noncoding RNA sequences were predicted based on the rRNA and Rfam database, respectively.

### Comparative genomic analysis

Gene family clustering was performed using Orthofinder (option: -M msa) [53] based on the protein sequences of 14 species. Common single-copy genes were used to construct a maximum likelihood phylogenetic tree using Randomized Axelerated Maximum Likelihood (RAxML) software (model: PROTGAMMAWAG) [54]. The divergence times of the selected species were estimated using the MCMCtree subroutine of Phylogenetic Analysis by Maximum Likelihood (PAML; option: nsample: 3000000, burn: 8000000, seqtype: 0, model: 4) [55]. The Computational Analysis of gene Family Evolution (CAFE) was used to predict the contraction and expansion of gene families. MCScanX (option: -a -e 1e^−5^ -s [5]) [56] was used to analyze the genome collinearity. The ratio of the number of nonsynonymous substitutions per nonsynonymous site/*K*s (*K*a/*K*s) values of the collinear gene pairs were calculated using the yn00 module in PAML.

### SV detection

The genome was aligned using MUMmer (version 4.0.0rc) [57] with the haplotype A used as reference. Subsequently, SyRI (version 1.5) [58] was used to detect and identify variations in the haplotype genome.

### Key enzymes in monoterpenoid biosynthesis

To identify the genes involved in monoterpenoid biosynthesis, a series of functional protein sequences were retrieved from the National Center for Biotechnology Information (Table S12). The related genes involved in monoterpene biosynthesis were identified in the *M. suaveolens* genome using Protein BLAST (BLASTP; option: e-value 1e^−10^). Subsequently, the phylogenetic trees of monoterpenoid biosynthetic genes were constructed and visualized using MEGA-X64 software with 1000 bootstrap replicates.

### RNA sequencing and volatile metabolite analysis by gas chromatography–mass spectrometry

Tissues of roots, leaves, stems of plants grown for 30, 120, 240 days were collected and frozen in liquid nitrogen for transcriptome and metabolome analyses. Thereafter, we extracted and isolated total RNA from those tissues. The qualified samples were used to build a library using the MGIEasy RNA Library Prep Kit for BGI®. An Agilent 2100 Bioanalyzer was used to detect the quality of the library, and DNBSEQ was used for RNA sequencing. Low-quality, irrelevant sequences were filtered using fastq [59]. The Spliced Transcripts Alignment to a Reference (STAR) was used to align clean data [60], and StringTie [61] was used to assemble the transcripts. The expression levels of each gene in terms of FPKM were generated using RNA-Sequencing by Expectation–Maximization (RSEM) [62].

A gas chromatograph (8890; Agilent) and a mass spectrometer (7000D; Agilent) were used for gas chromatography–mass spectrometry analysis. DB-5MS (5% phenyl-polydimethylsiloxane) capillary column (Agilent) was used for the separation of VOCs, the line speed was 1.2 mL/min. The injector temperature was kept at 250°C, the detector at 280°C, and the solvent delay was 3.5 minutes. The oven temperature was programmed from 40°C (3.5 minutes), increasing at 10°C/min to 100°C, at 7°C/min to 180°C, at 25°C/min to 280°C, and held for 5 min. The mass spectrometer was operated under the following conditions: electron impact mode, 70 eV, four-stage rod temperature, ion source and mass spectrometry interface set at 150°C, 230°C, and 280°C. Accurate scanning of qualitative and quantitative ions was performed in the ion detection mode (selected ion monitoring), and MassHunter was used for data analysis.

### Co-expression network construction

An Hidden Markov Model (HMM) search (*NTF2*, PF02136 and *CYP450*, PF00067) combined with BLASTP was performed to identify candidate *IPGI* and *CYP71D* genes in the *M. suaveolens* genome. The protein data of *IPGI* and *CYP71D* genes with known function are listed in Tables S15 and S16. The phylogenetic trees of *IPGI* and *CYP71D* genes were constructed and visualized using MEGA-X64 software with 1000 bootstrap replicates. The heatmap was generated using expression data to visualize the levels of *NTF2* and *CYP71D* genes in different tissues. Correlation analysis was used to evaluate *NTF2* and *CYP71D* genes exhibiting high correlations with monoterpene biosynthesis genes and metabolites. The co-expression network was visualized using Cytoscape. The cutoff values were set as follows: FPKM values of the tested gene >1, *p*-value of the correlation test <0.05, and Pearson correlation coefficient (*r*) >0.8. Of note, the *NTF2* protein sequence used in this study was derived from bacteria; hence, the parameter settings were adjusted (i.e. *r* > 0.6).

## Acknowledgements

This work was supported by introduces the talented person scientific research start funds subsidization project of Chengdu University of Traditional Chinese Medicine (030040015, 030040017) and Hubei science and technology planning project (2020BCB038).

## Author Contributions

H.Y., C.S., L.L., C.W., and S.C conceived and supervised the study. H.Y., and G.Z. prepared the materials. Z.W., L.Y., H.T., G. C., and X.K. performed genome assembly and annotation. T.H., H.Z., X.T., and Z.L. analyzed the data. X.T., H.Y., Y.W., and Y.Z. drew the figures. H.Y., S.Z., C.S., L.L., and C.W wrote the manuscript. All authors read and approved the final manuscript.

## Data availability statement

The genome data of *M. suaveolens* were uploaded in 1 K Medicinal Plant Genome Database (http://www.herbgenome.com) [63]. RNA-seq data of different tissues were available in NCBI under Biological Project ID (PRJNA938973, https://dataview.ncbi.nlm.nih.gov/object/PRJNA938973?reviewer=mp56u3u4kcflnb7g3q0gum9qmg).

## Conflict of interests 

The authors declare that they have no conflict of interest.

## Supplementary information

Supplementary data is available at *Horticulture Research* online.

## Supplementary Material

Web_Material_uhae022
