# Supplementary material for: A haplotype-resolved gap-free genome assembly provides novel insight into monoterpenoid diversification in Mentha suaveolens ‘Variegata’
Source: Hortic Res. 2024 Jan 17;11(3):uhae022. doi: 10.1093/hr/uhae022 (PMC10925848; doi:10.1093/hr/uhae022)
Supplement: Web_Material_uhae022 [file web_material_uhae022.zip › Supplementary Materials.docx]

**Supplementary Materials**


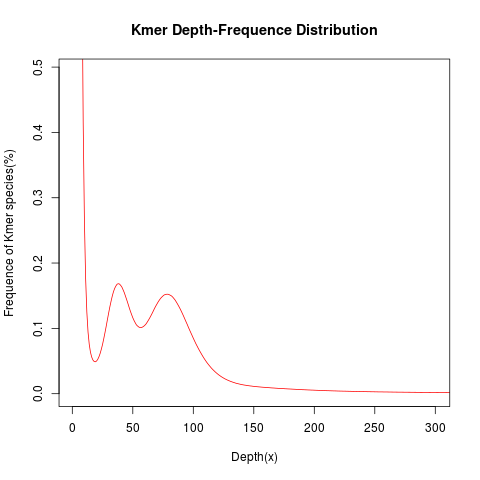


Figure. S1 The 19-mer distribution of Kmer-Depth and Kmerspecies-Frequence.


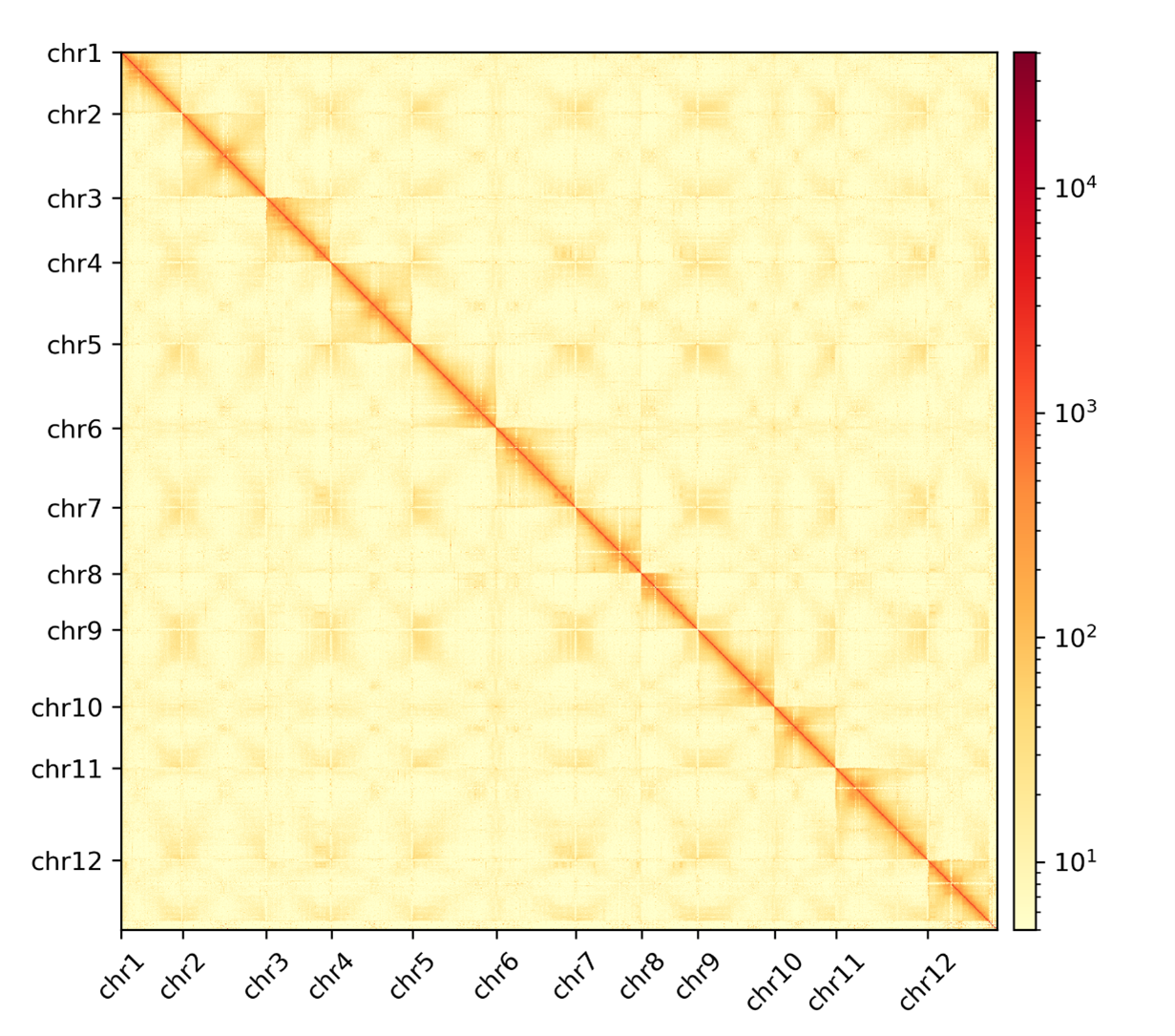

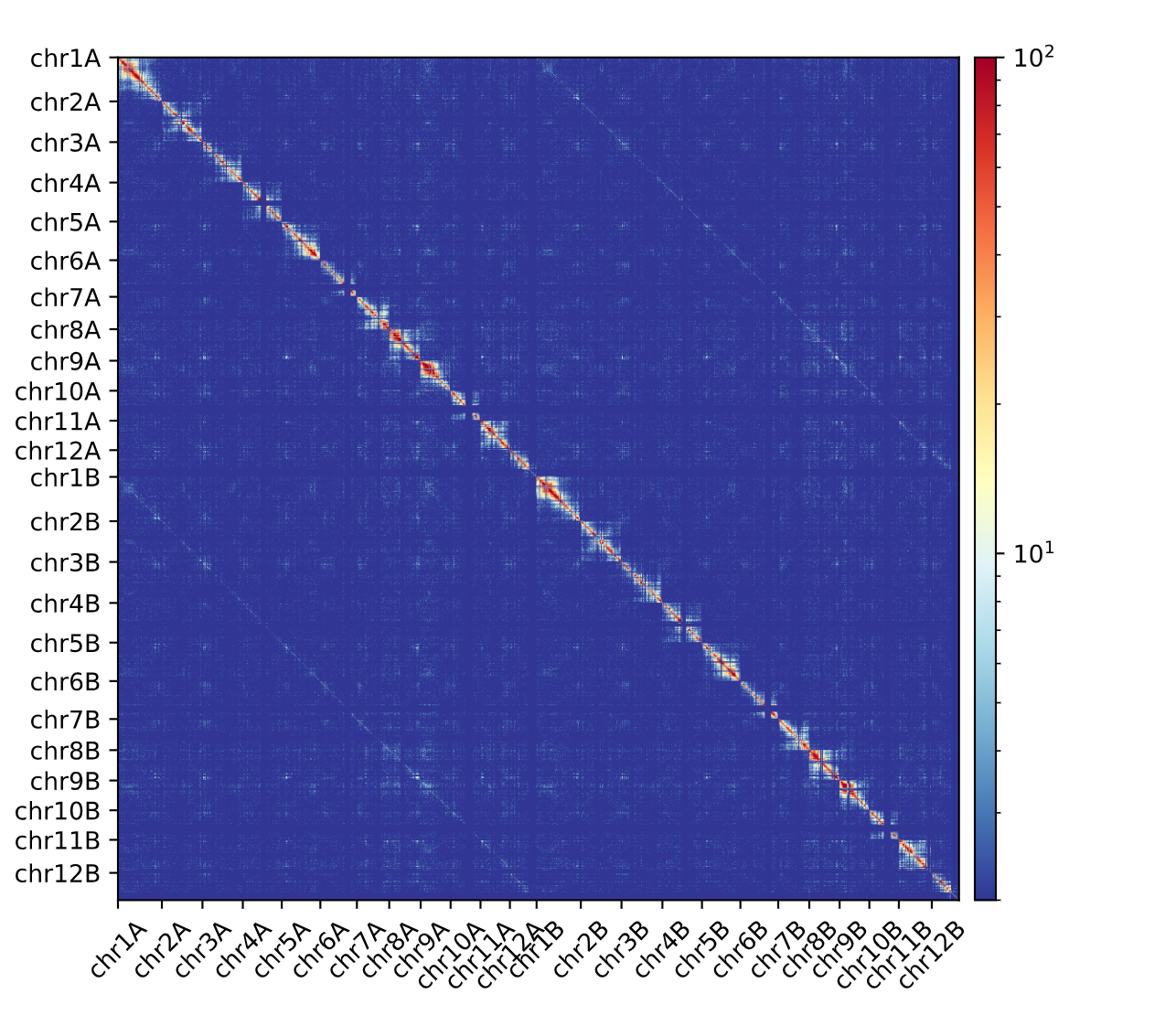


Figure. S2 Hi-C chromatin interaction map of the pineapple mint assembly.


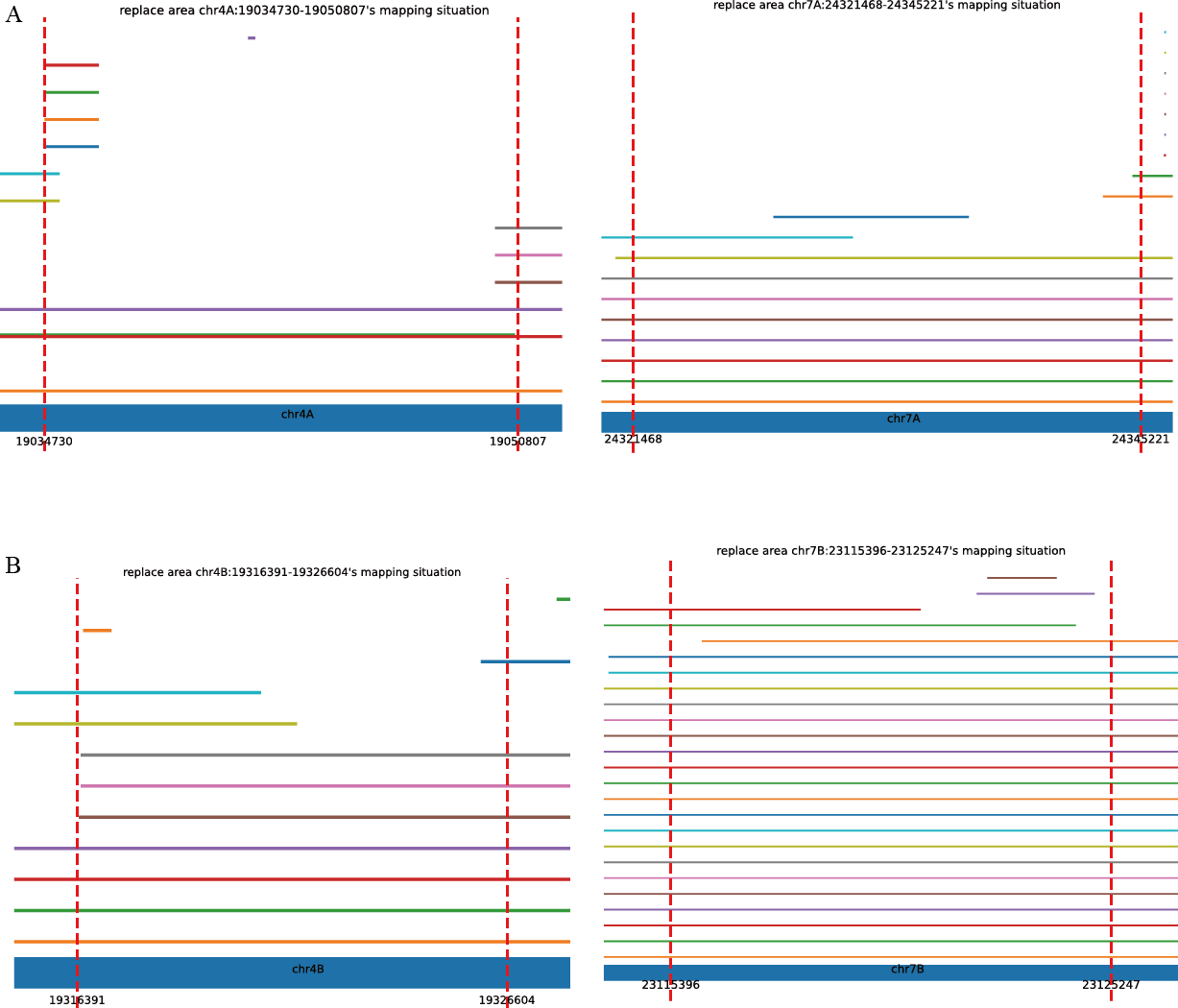


Figure. S3 Gap filling verification results.


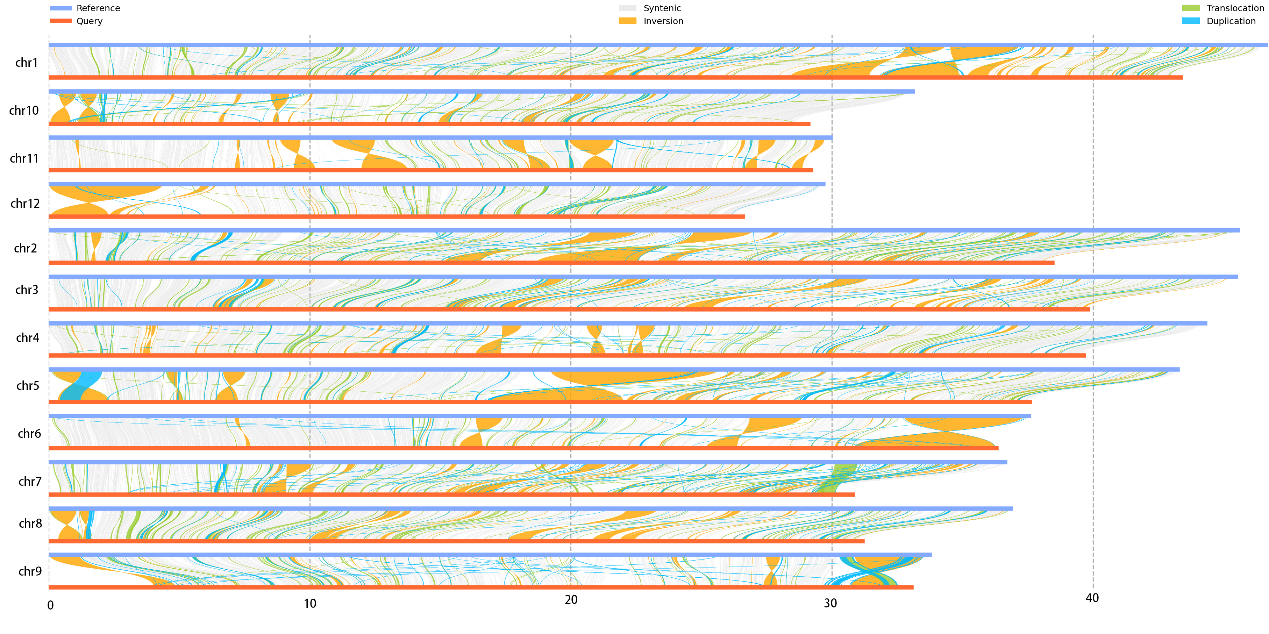


Figure. S4 Collinearity between the *M. suaveolens* and *M. longifolia* (CMEN 585) genomes. blue chromosome: *M. longifolia* (CMEN 585), orange chromosome: *M. suaveolens*.


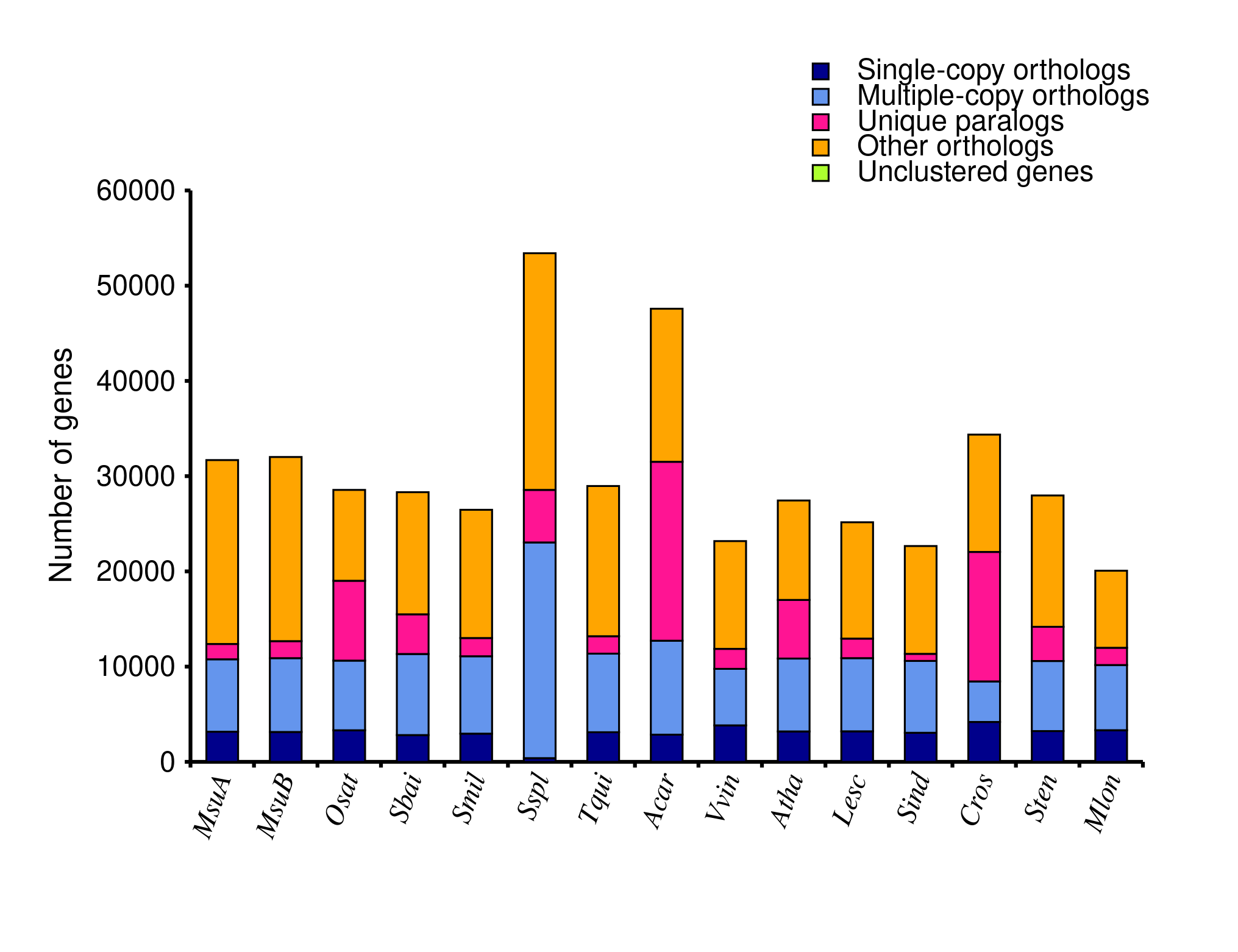


Figure. S5 Distribution of genes and gene families of 14 plant species we investigated. MsuA: *M. suaveolens* HapA, MsuB: *M. suaveolens* HapB, Osat: *O. sativa*, Sbai: *S. baicalensis*, Smil: *S. miltiorrhiza*, Sspl: *S. splendens*, Tqui: *T. quinquecostatus*, Acar: *A. carvifolia*, Vvin: *V. vinifera*, Atha: *Arabidopsis thaliana*, Lesc: *L. esculentum*, Sind: *S. indicum,* Cros: *C. roseus,* Sten: *Schizonepeta tenuifolia,* Mlon: *M. longifolia.*


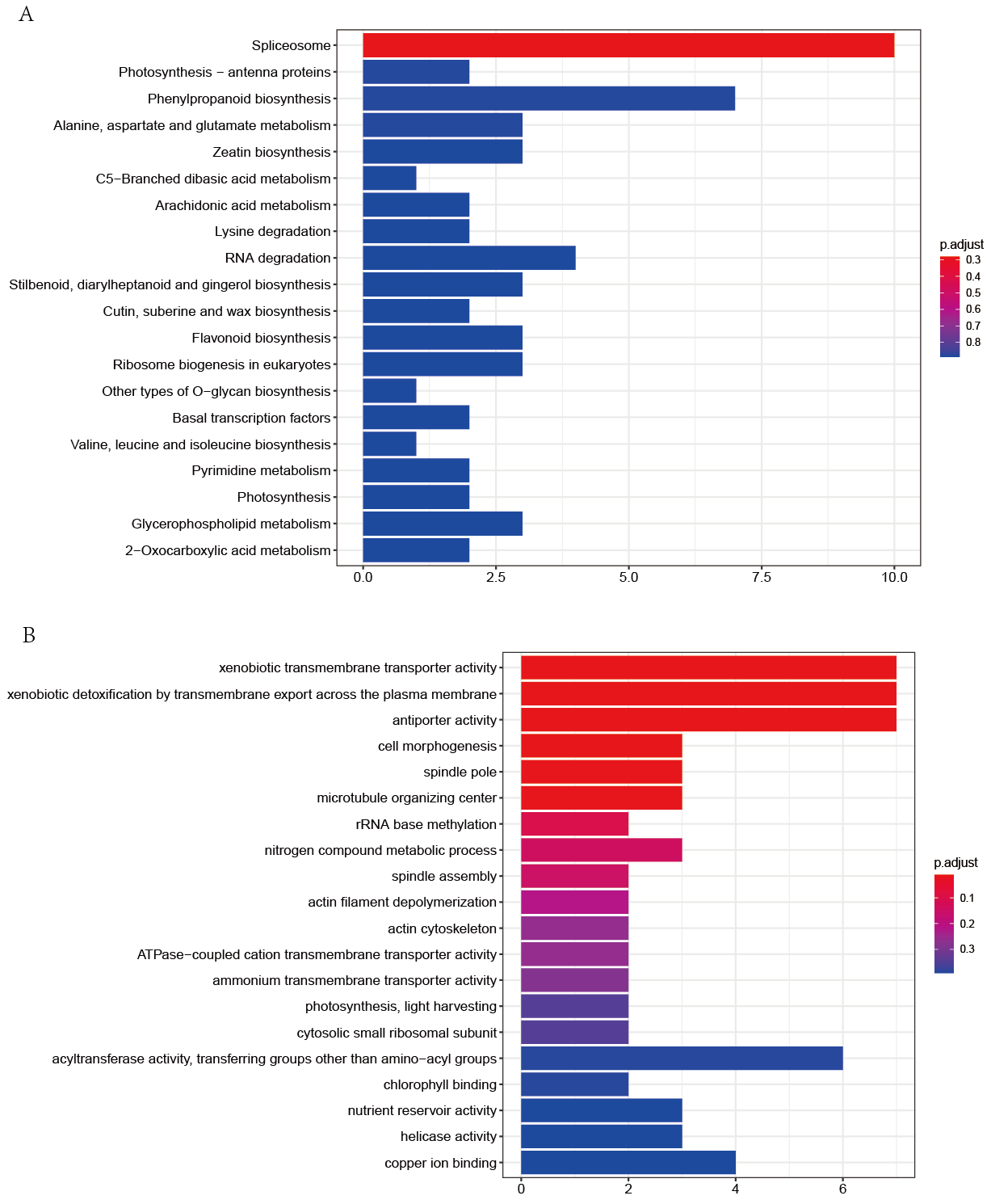


Figure. S6 KEGG and GO analysis of uniq gene in all species. (A). KEGG analysis of uniq gene in *M. suaveolens* genome. (B) GO analysis of uniq gene in *M. suaveolens* genome.


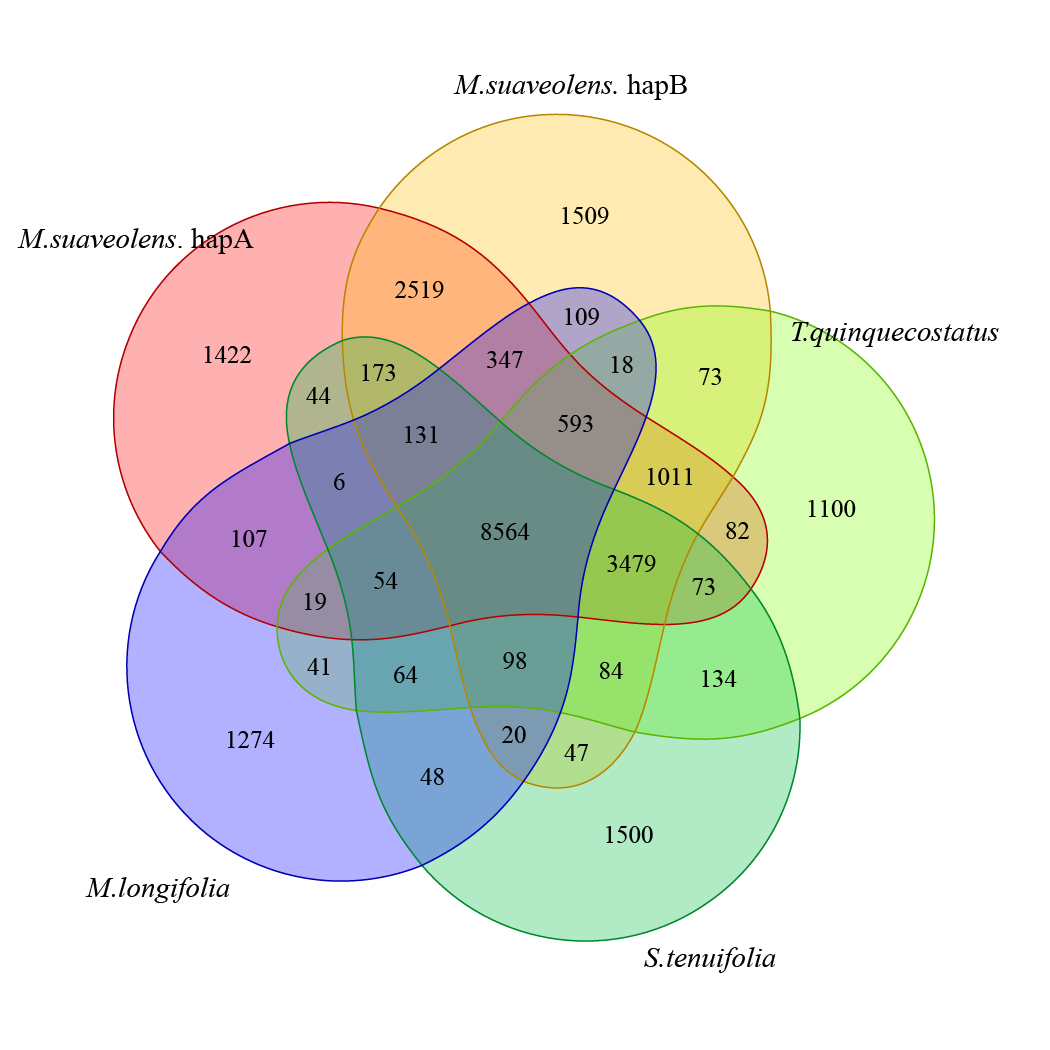


Figure. S7 Statistical venn diagram of gene family clustering results


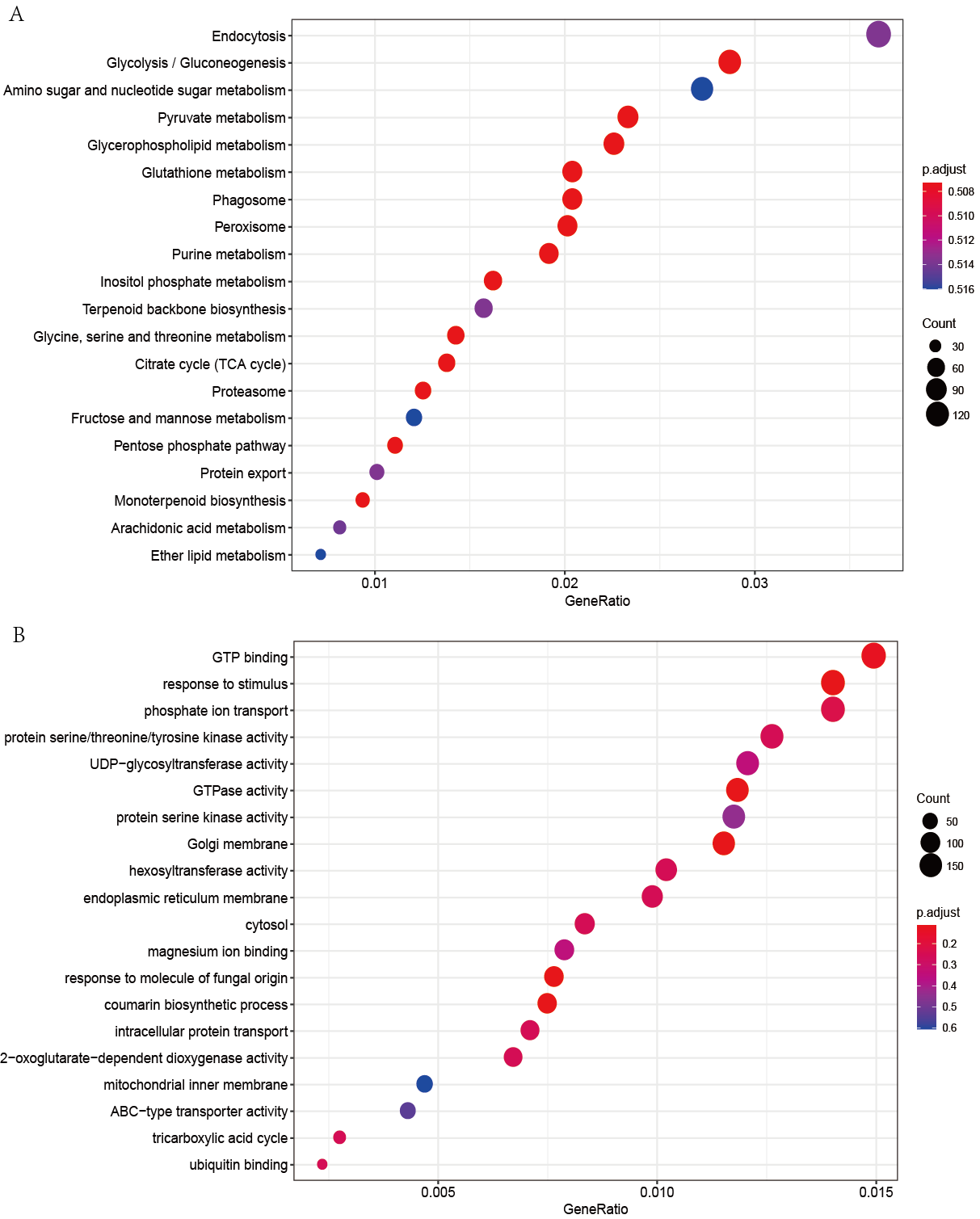


Figure. S8 KEGG and GO analysis of share gene in fourteen species. (A). KEGG analysis of share gene in *M. suaveolens* genome. (B). GO analysis of share gene in *M. suaveolens* genome.


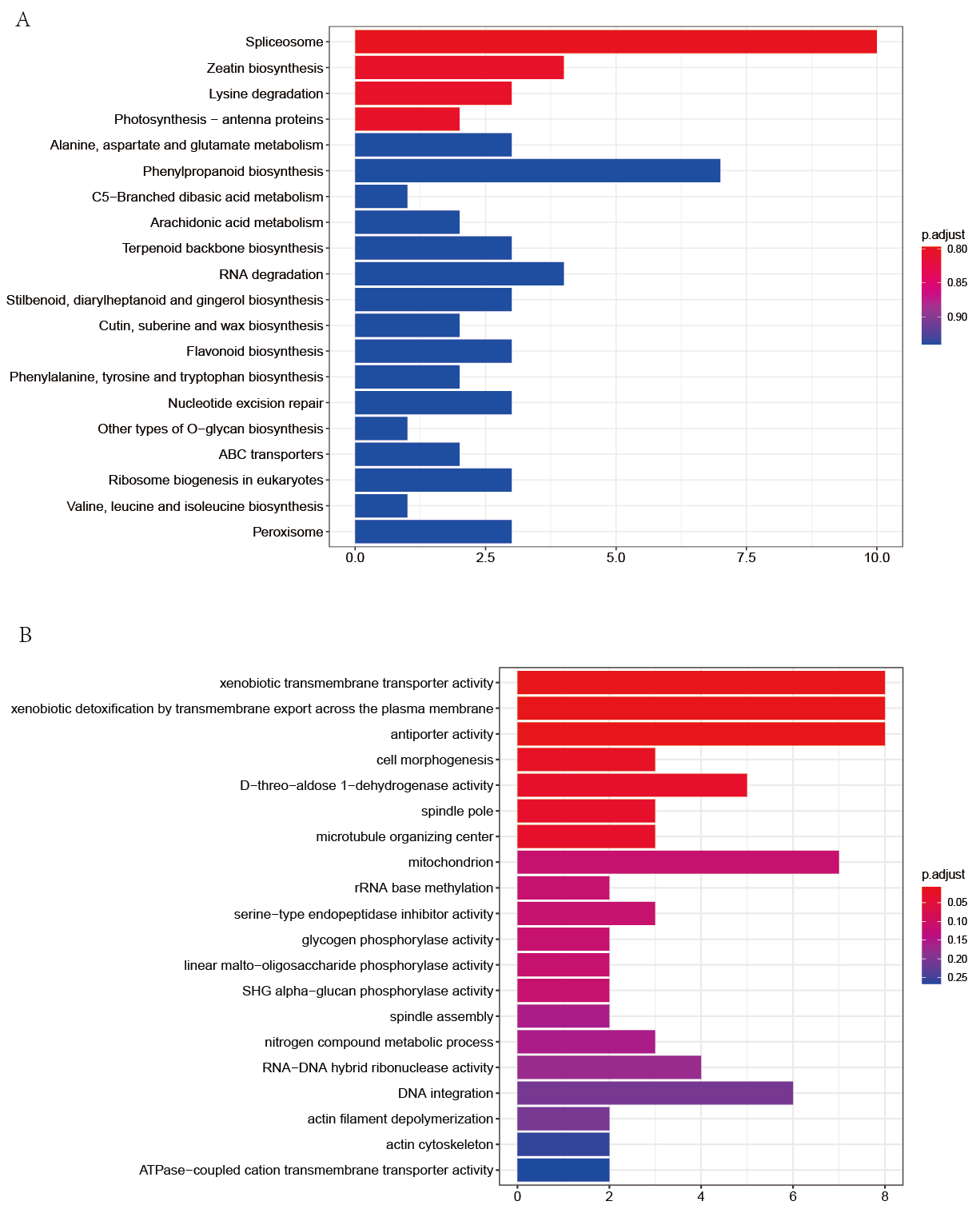


Figure. S9 KEGG and GO analysis of unique gene in fourteen species. (A). KEGG analysis of uniq gene in *M. suaveolens* genome. (B). GO analysis of uniq gene in *M. suaveolens* genome.


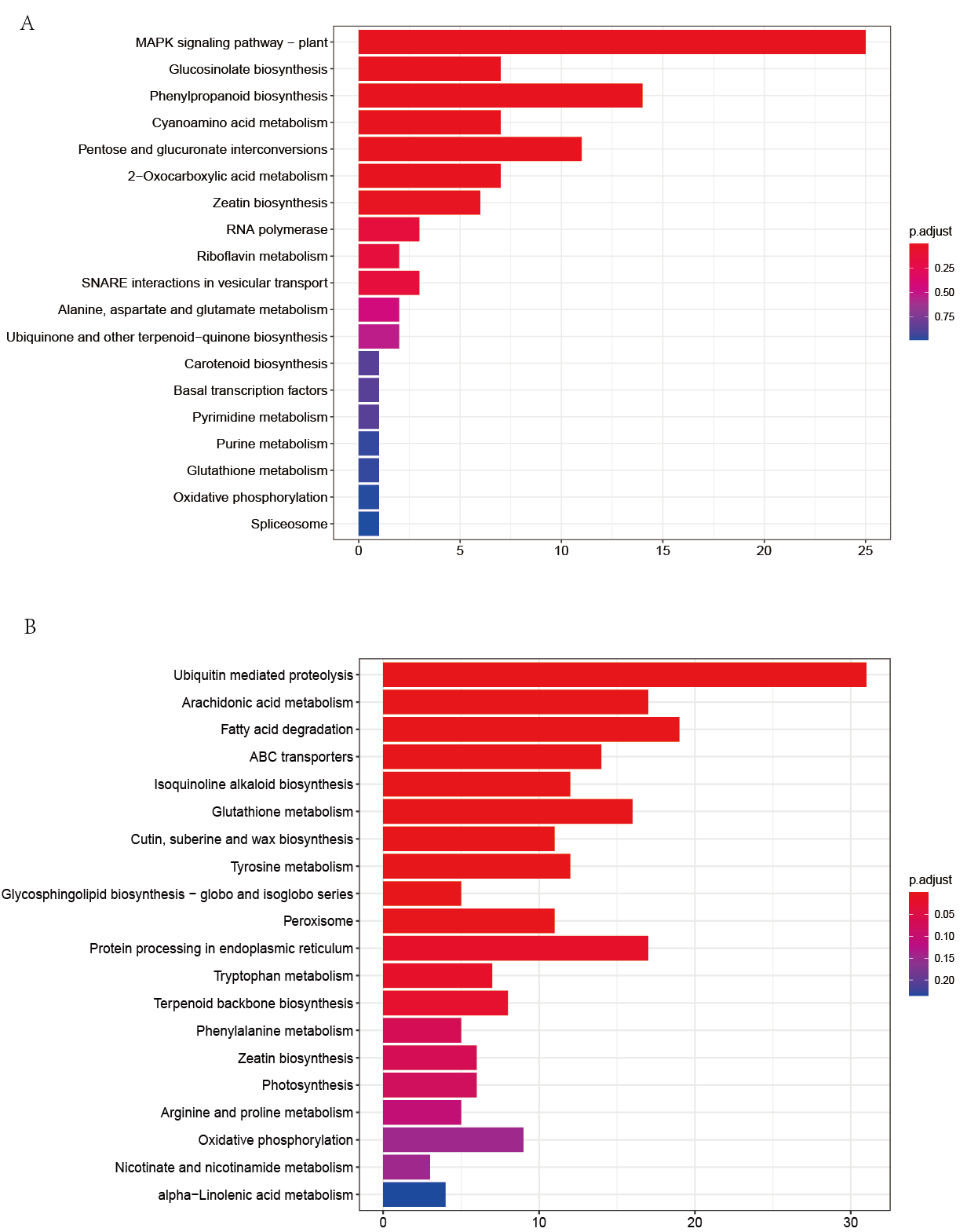


Figure. S10 KEGG analysis of contracting and expanding gene family in *M. suaveolens* genome. (A). KEGG analysis of contracting gene family. (B). KEGG analysis of expanding gene family.


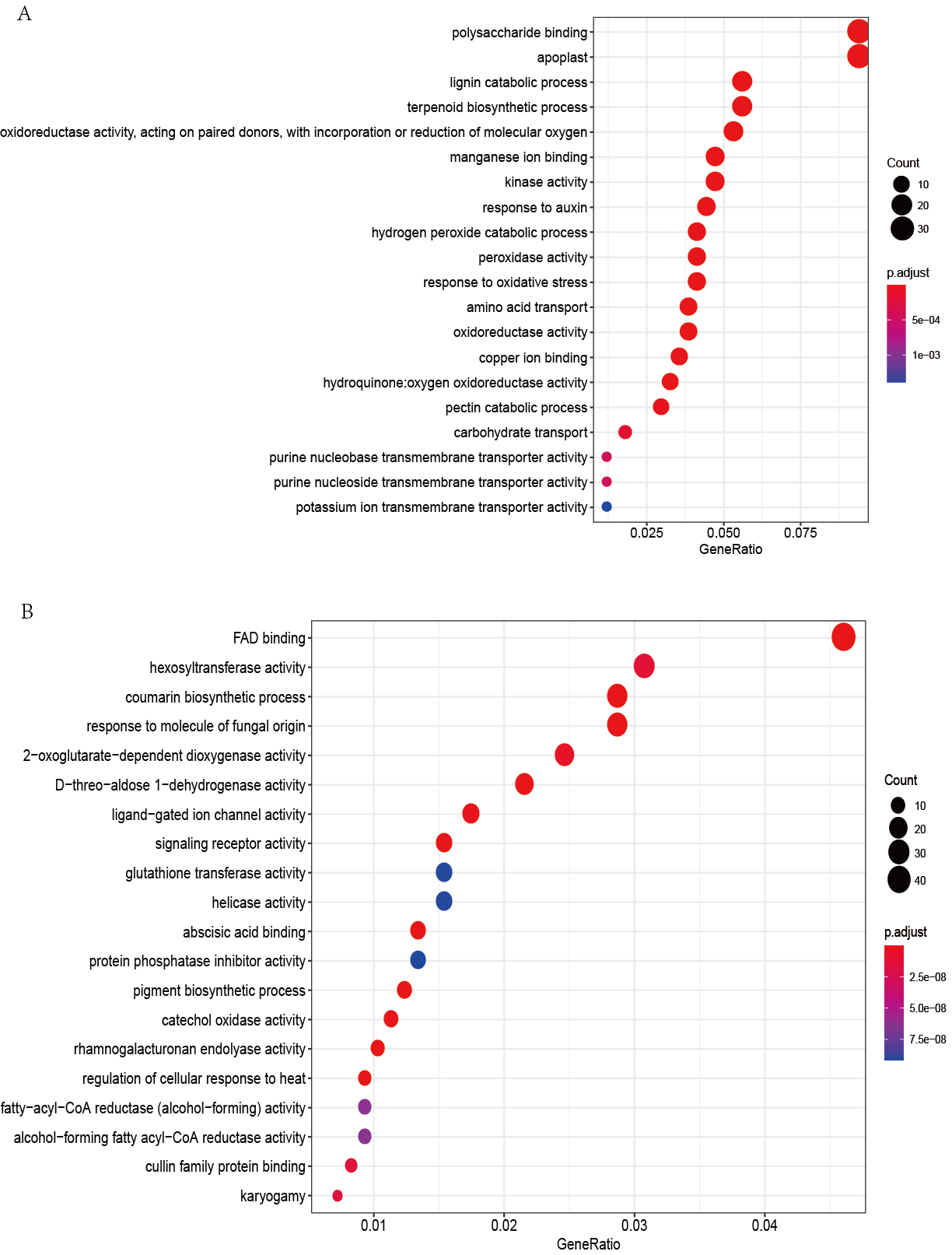


Figure. S11 GO analysis of contracting and expanding gene family in *M. suaveolens* genome. (A). GO analysis of contracting gene. (B). GO analysis of expanding gene.


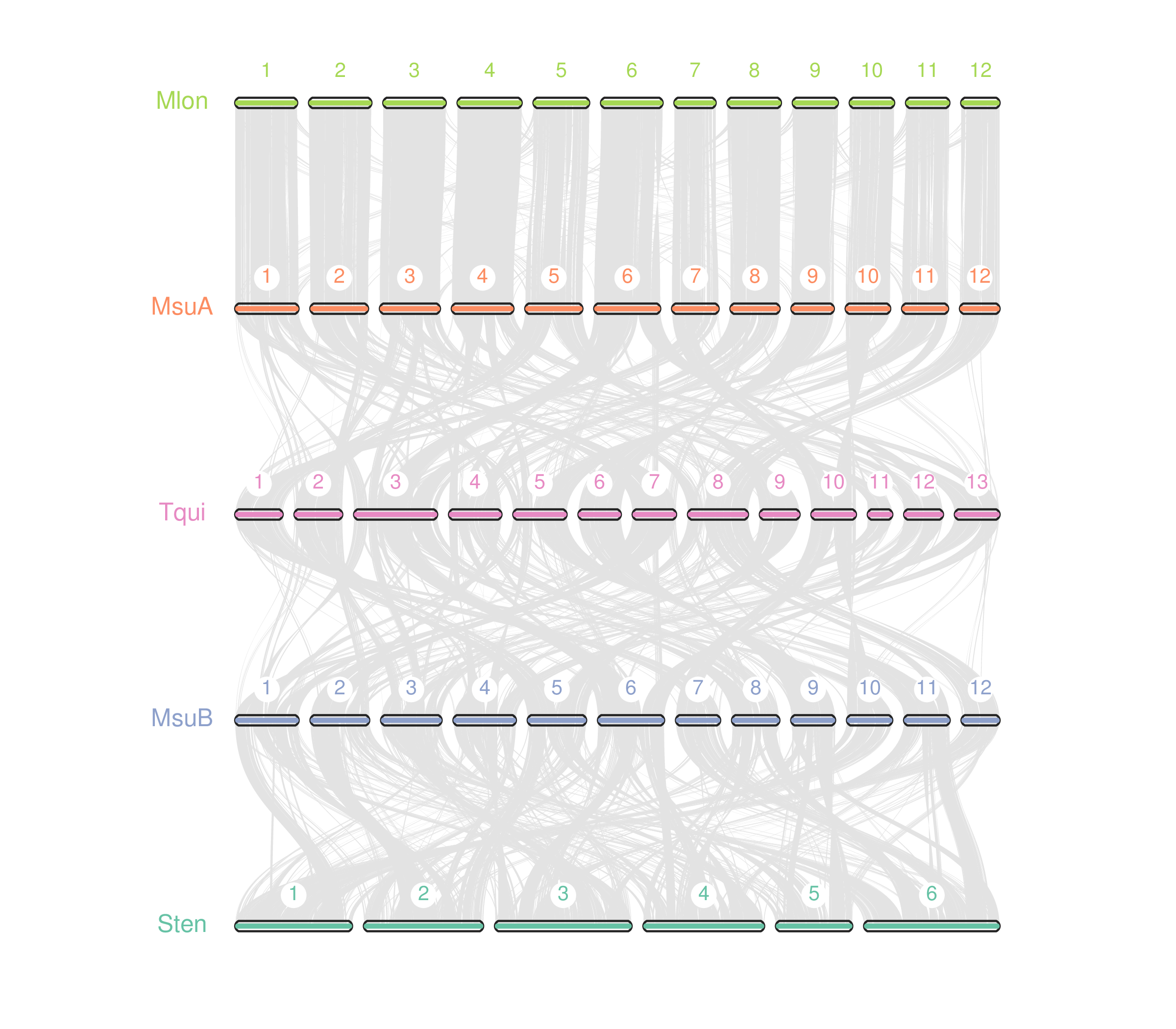
Figure. S12 Syntenic plots. The thick colored bars of different colors represent the chromosomes of each species, and the gray lines indicate collinear blocks between the two ends of the line. Msua: *M. suaveolens*, Mlon: *M. Longifolia*, and Tqui: *T. quinquecostatus*.

**
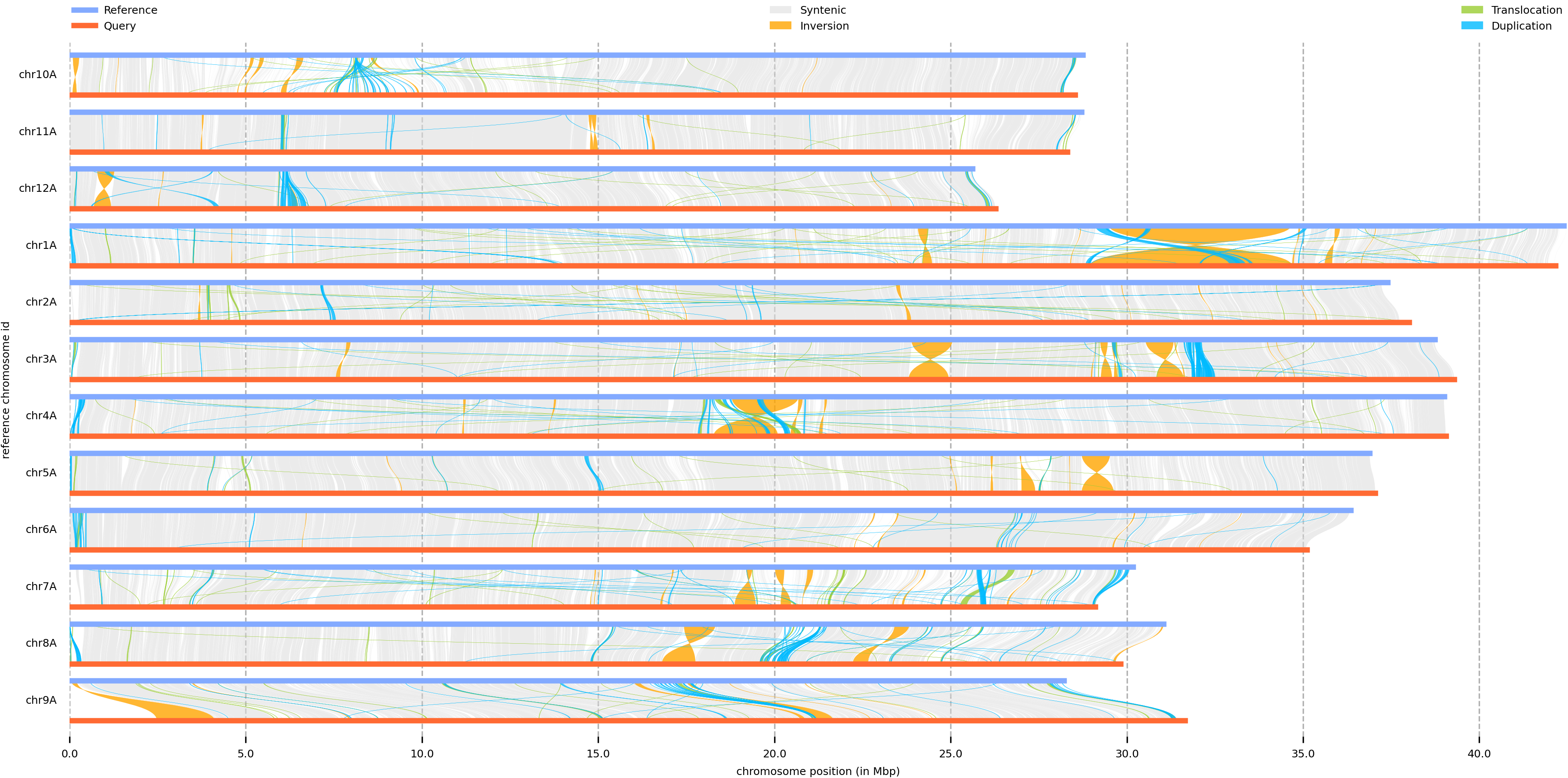
**

Figure. S13 Whole-genome level structural variation (SV) display. Collinearity between hapA and hapB genomes. blue chromosome: hapA; orange chromosome: hapB.


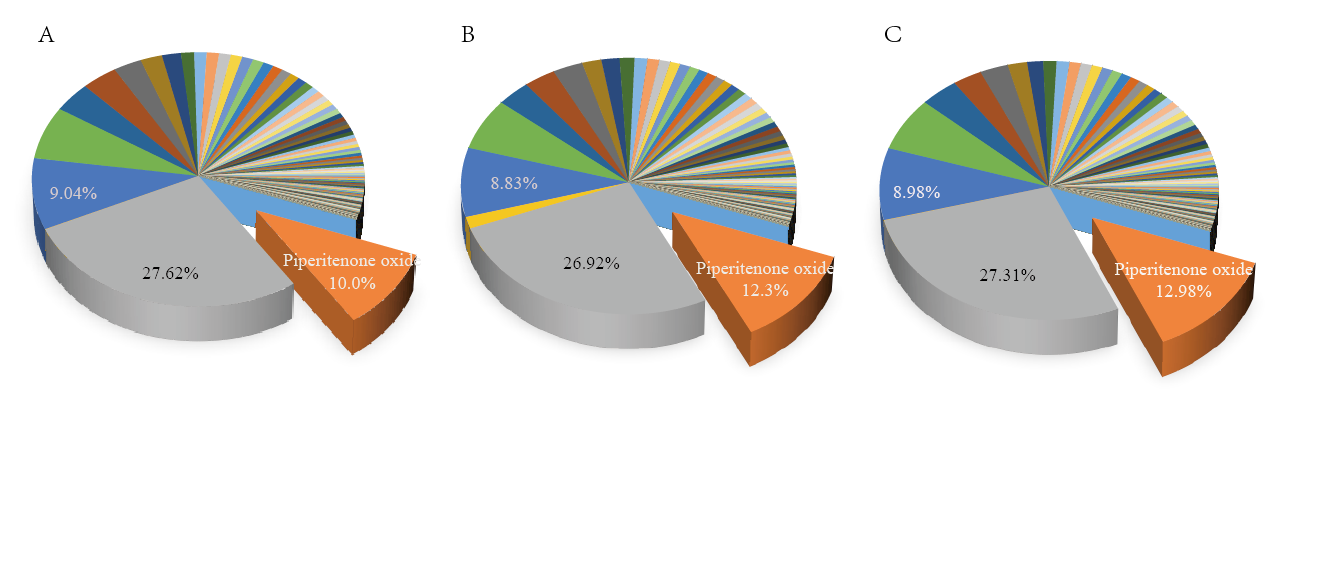


Figure. S14 The analysis of volatile metabolites in *M. suaveolens.* (A). Classification of all volatile metabolites of 30 day. (B). Classification of all volatile metabolites of 120 day. (C). Classification of all volatile metabolites of 240 day.


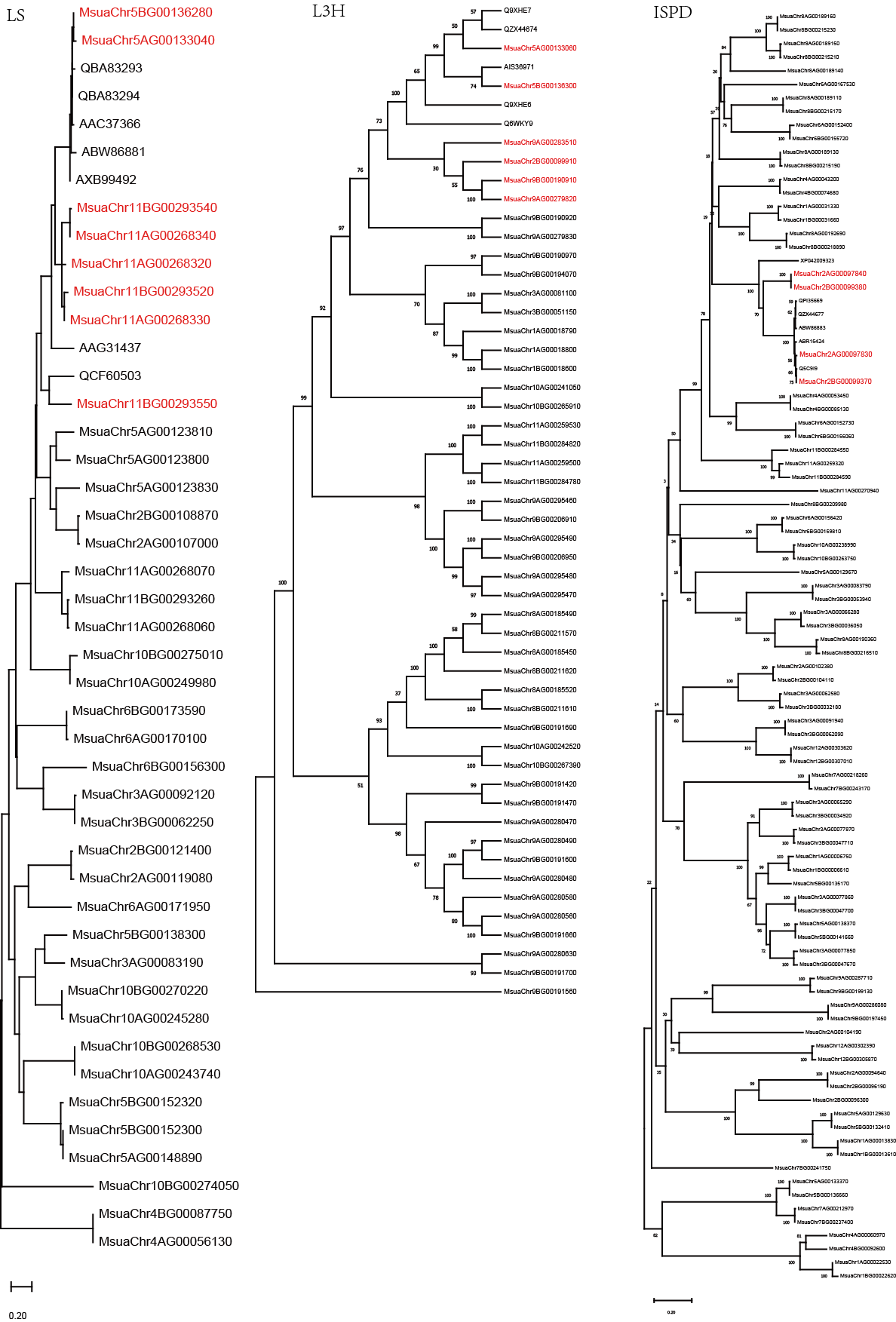


Figure. S15 Phylogenetic tree of genes involved in menthol monoterpene biosynthesis using MEGA-X64 with 1000 bootstrap replicates by Neighbor-joining (NJ) method. Candidate genes are highlighted in red.


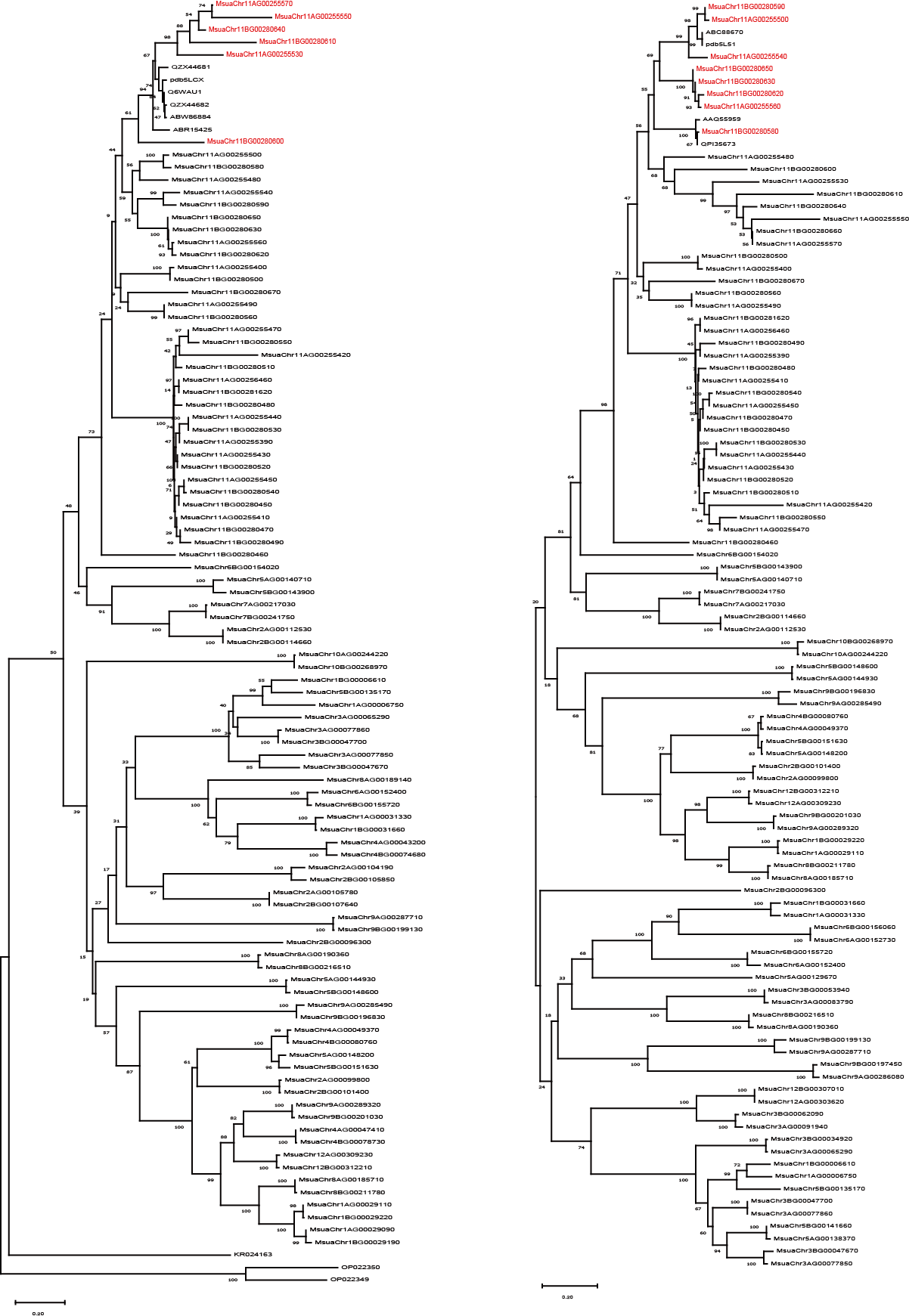


Figure. S16 Phylogenetic tree of *ISPR* and *MR* involved in *M. suaveolen*. Candidate genes are highlighted in red.


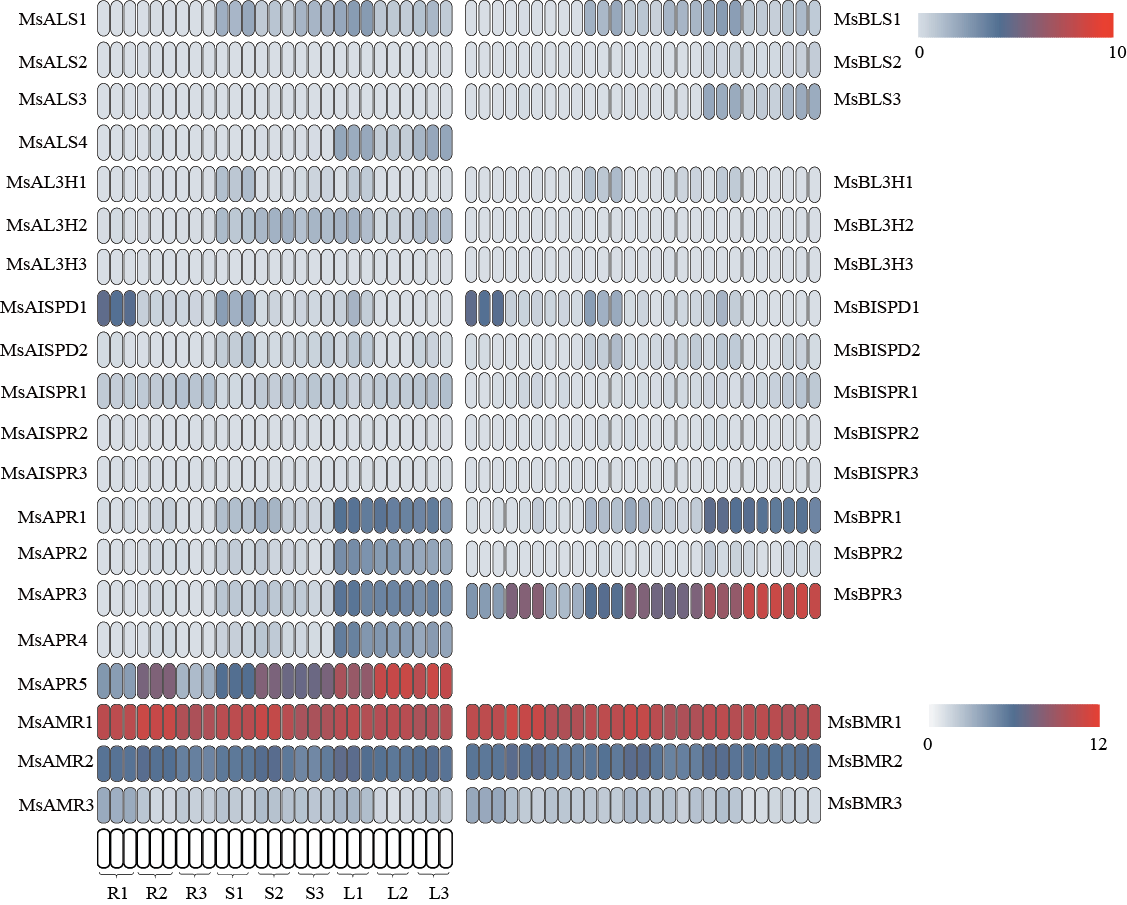


Figure. S17 Expression profiles of identified genes in *M. suaveolens*. The expression of each identified gene is represented as the FPKM of *M. suaveolens* transcriptomes. Sample sets 1, 2, 3, represent three growth stages (30, 120, 240 day) and three biological replicates.


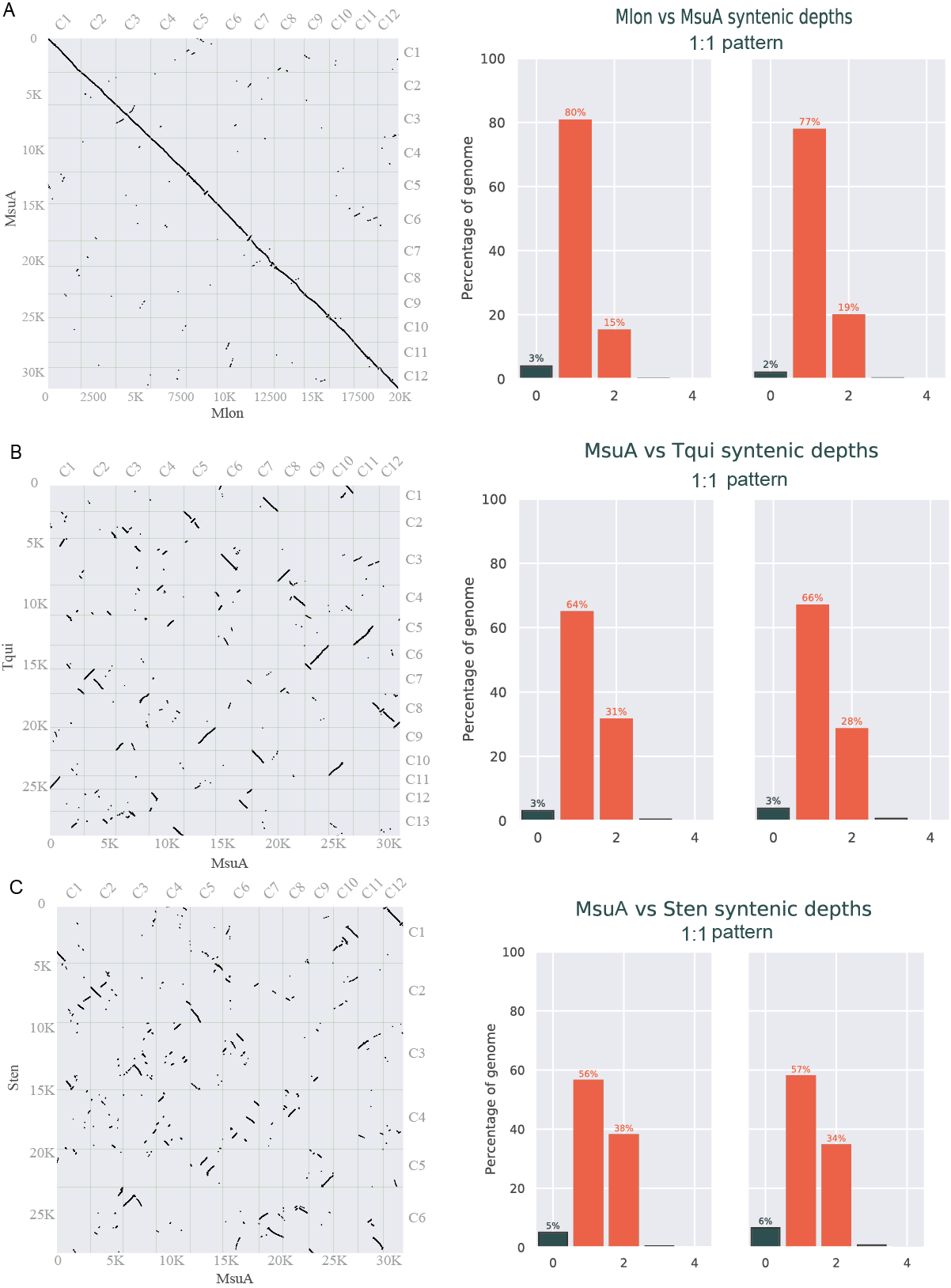


Figure. S18 Dotplot of syntenic blocks and depths in the *M. suaveolens* and *M. longifolia* and *M. suaveolens* hapA with *S. tenuifolia* genome. (A) Dotplot of syntenic blocks and depths in the *M. suaveolens* and *M. longifolia.* (B) Dotplot of syntenic blocks and depths in the *M. suaveolens* and *T. quinquecostatus*. (C) Dotplot of syntenic blocks and depths in the *M. suaveolens* and *S. tenuifolia.*


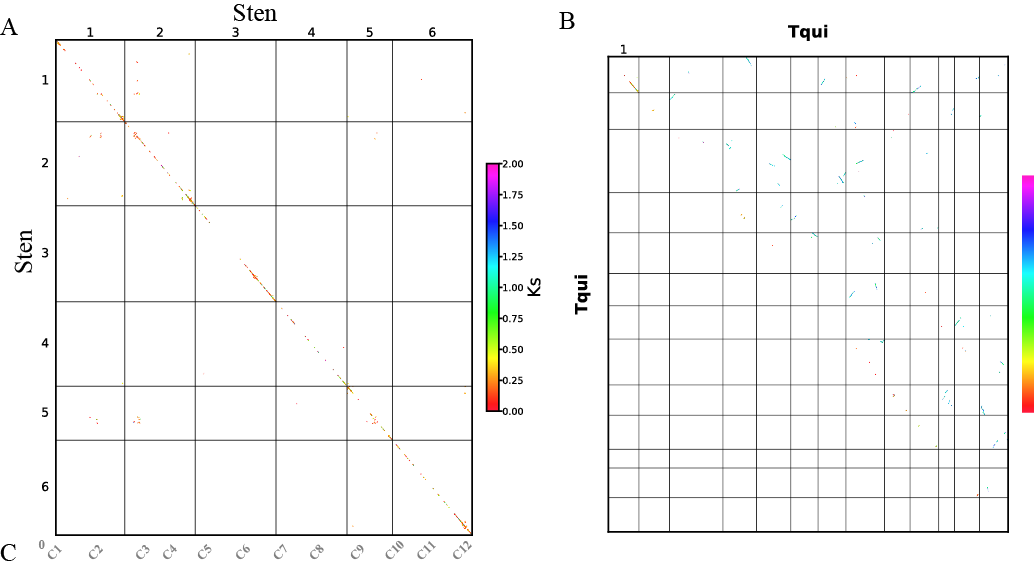


Figure. S19 Collinear block of Ks=0~0.5 area in *S. tenuifolia* (Sten), *T. quinquecostatus* (Tqui)*.* (A) Collinear block of Ks=0~0.5 area in *S. tenuifolia*. (B) Collinear block of Ks=0~0.5 area in *T. quinquecostatus.*

Table. S1 Summary statistics of ONT, DNBSEQ and HiC sequencing

|  | ***Mentha suaveolens*** |
| --- | --- |
| Oxford Nanopore sequencing data |  |
| Total base (Gb) | 29.73 |
| Total reads | 630,987 |
| Maximum length (bp) | 427,928 |
| Average length (bp) | 47,113 |
| N50 (bp) | 54,513 |
| Hi-C sequencing data |  |
| Clean base (Gb) | 71.2 |
| Clean reads (bp) | 477,168,056 |
| Q20 (%) | 98.630% |
| Q30 (%) | 94.823% |
| PacBio HiFi data |  |
| Total Bases(Gb) | 31.27 |
| Reads length N50(bp) | 18,322 |
| Assembly data |  |
| Mapping rate (%) | 97.17 |
| Coverage at least 20X(%) | 99.44 |
| Genome BUSCOs (C) | 99.1 |
| Qv | 46.45 |

Table. S2 Telomere repeat sequence and centromere prediction regional location in haplotype-resolved genome

|  | **Telomere** | | **Centromere** | |
| --- | --- | --- | --- | --- |
| **chr** | **upstream** | **downstream** | **start** | **end** |
| chr1A | 0 | 764 | 32,800,005 | 33,610,000 |
| chr2A | 168 | 2,324 | 21,400,165 | 21,949,744 |
| chr3A | 2,560 | 799 | 31,604,489 | 32,010,000 |
| chr4A | 2,505 | 2,460 | 18,500,001 | 19,398,898 |
| chr5A | 578 | 937 | 27,152,501 | 27,728,751 |
| chr6A | 1,067 | 2,533 | 26,600,001 | 27,449,423 |
| chr7A | 2,046 | 2,518 | 19,200,006 | 19,299,424 |
| chr8A | 950 | 509 | 20,203,417 | 21,434,074 |
| chr9A | 1,505 | 2,558 | 16,400,350 | 17,569,935 |
| chr10A | 2,559 | 2,247 | 8,000,014 | 8,427,034 |
| chr11A | 2,501 | 2,542 | 9,000,001 | 9,349,937 |
| chr12A | 0 | 564 | 5,854,853 | 6,290,424 |
| chr1B | 2,358 | 2,452 | 29,900,512 | 30,471,948 |
| chr2B | 2,556 | 697 | 21,800,008 | 22,289,995 |
| chr3B | 707 | 500 | 31,900,001 | 32,410,000 |
| chr4B | 2,538 | 2,557 | 19,400,001 | 20,298,066 |
| chr5B | 2,050 | 1,461 | 26,901,555 | 27,298,285 |
| chr6B | 2,419 | 2,542 | 26,004,858 | 26,849,338 |
| chr7B | 2,547 | 1,606 | 19,000,001 | 19,367,366 |
| chr8B | 2,019 | 1,265 | 19,600,001 | 20,331,646 |
| chr9B | 255 | 1,040 | 21,057,359 | 21,429,815 |
| chr10B | 2,551 | 2,056 | 7,400,065 | 9,749,595 |
| chr11B | 2,559 | 2,559 | 8,902,251 | 9,299,407 |
| chr12B | 0 | 1,600 | 5,800,421 | 67,98,392 |

Table. S3 Assessment of gap number, QV value, and contig number of each chromosome in haplotype-resolved genome

| Chr | Length (bp) | Gap | QV | Contig number |
| --- | --- | --- | --- | --- |
| chr1A | 42,165,926 | 0 | 45.6400 | 1 |
| chr2A | 37,394,926 | 0 | 46.9373 | 1 |
| chr3A | 38,745,243 | 0 | 46.5363 | 1 |
| chr4A | 39,005,997 | 0 | 46.0924 | 1 |
| chr5A | 37,045,531 | 0 | 46.2286 | 1 |
| chr6A | 35,112,294 | 0 | 46.6420 | 1 |
| chr7A | 30,179,724 | 0 | 47.8569 | 1 |
| chr8A | 31,046,934 | 0 | 44.2324 | 1 |
| chr9A | 28,219,553 | 0 | 46.6368 | 1 |
| chr10A | 28,745,801 | 0 | 47.7112 | 1 |
| chr11A | 28,709,128 | 0 | 46.8573 | 1 |
| chr12A | 25,616,839 | 0 | 47.3592 | 1 |
| chr1B | 42,483,916 | 0 | 46.1494 | 1 |
| chr2B | 38,012,915 | 0 | 46.2065 | 1 |
| chr3B | 39,288,234 | 0 | 46.8392 | 1 |
| chr4B | 39,053,201 | 0 | 46.1741 | 1 |
| chr5B | 36,893,854 | 0 | 45.9558 | 1 |
| chr6B | 36,357,466 | 0 | 46.9794 | 1 |
| chr7B | 29,106,550 | 0 | 47.7691 | 1 |
| chr8B | 29,826,324 | 0 | 45.0769 | 1 |
| chr9B | 31,646,362 | 0 | 47.0989 | 1 |
| chr10B | 28,532,217 | 0 | 47.9877 | 1 |
| chr11B | 28,313,633 | 0 | 46.8959 | 1 |
| chr12B | 26,284,060 | 0 | 46.1895 | 1 |

Table. S4 Summary of repeat contents.

|  | HapA | | | HapB | |
| --- | --- | --- | --- | --- | --- |
| Type | Length (bp) | % in genome | Length (bp) | | % in genome |
| DNA | 19,439,693 | 4.82 | 19,354,279 | | 4.79 |
| LINE | 4,399,711 | 1.09 | 4,192,426 | | 1.04 |
| SINE | 9,030 | 0 | 9,895 | | 0 |
| LTR | 137,470,926 | 34.08 | 136,530,580 | | 33.76 |
| LTR-Gypsy | 79,484,454 | 19.7 | 78,985,989 | | 19.53 |
| LTR-Copia | 40,419,836 | 10.02 | 40,659,886 | | 10.05 |
| Satellite | 238,043 | 0.06 | 167,619 | | 0.04 |
| Simple repeat | 70,222 | 0.02 | 70,870 | | 0.02 |
| Other | 14,078 | 0 | 11,483 | | 0 |
| Unknown | 95,612,698 | 23.7 | 99,272,522 | | 24.55 |
| Total | 249,298,078 | 61.8 | 251,413,845 | | 62.17 |

Table. S5 Basic statistics of all evidence sets in HapA genome

| Method | Species | Gene number | Average gene length (bp) | Average CDS length (bp) | Average exon per gene | Average exon length (bp) | Average intron length (bp) |
| --- | --- | --- | --- | --- | --- | --- | --- |
| Ab initio GlimmmerHMM |  | 55,318 | 6,380.42 | 939.55 | 3.83 | 245.04 | 1,919.72 |
| Ab initio AUGUSTUS |  | 36,553 | 2,793.26 | 1,238.94 | 5.33 | 232.64 | 359.34 |
| Homology-based | *Scutellaria baicalensis* | 49,018 | 12,965.35 | 869.42 | 3.43 | 253.15 | 4,968.64 |
| Homology-based | *Salvia miltiorrhiza* | 48,263 | 13,992.47 | 903.95 | 3.58 | 252.43 | 5,071.02 |
| Homology-based | *Mentha longifolia* | 88,946 | 9,732.02 | 649.22 | 2.71 | 239.24 | 5,300.35 |
| Homology-based | *Salvia splendens* | 50,405 | 12,398.37 | 952.43 | 3.69 | 258.28 | 4,258.75 |
| RNAseq |  | 19,629 | 4,510.64 | 1,100.09 | 5.84 | 333.84 | 529.98 |
| Integration |  | 31,368 | 4,403.20 | 1,195.05 | 5.29 | 272.22 | 689.67 |
| Final set |  | 31,688 | 3,957.64 | 1,164.46 | 5.12 | 292.57 | 594.53 |

Table. S6 Summary of predicted genes.

| **Item** | **Number in HapA genome** | **Number in HapB genome** |
| --- | --- | --- |
| The total number of gene | 31,688 | 32,011 |
| The average of mRNA_length (bp) | 3,957.64 | 3,905.05 |
| The average cds_length of per gene (bp) | 1,164.46 | 1,151.68 |
| The average exon_number of per gene | 5.12 | 5.08 |
| The average of exon_length (bp) | 292.57 | 291.87 |
| The average of intron_length (bp) | 594.53 | 590.27 |
| The total number of exon | 162,258 | 162,757 |
| The total number of intron | 130,570 | 130,746 |
| The total intron length (bp) | 77,627,224 | 77,175,495 |

Table. S7 Genome annotation quality assessment using BUSCO in HapA genome.

|  | Assembly | | Annotation | |
| --- | --- | --- | --- | --- |
|  | Proteins | Percentage (%) | Proteins | Percentage (%) |
| Complete BUSCOs | 1,593 | 98.7 | 1,598 | 99.0 |
| Complete Single-Copy BUSCOs | 1,543 | 95.6 | 1,544 | 95.7 |
| Complete Duplicated BUSCOs | 50 | 3.1 | 54 | 3.3 |
| Fragmented BUSCOs | 5 | 0.3 | 0 | 0 |
| Missing BUSCOs | 16 | 1 | 16 | 1 |
| Total BUSCO groups searched | 1,614 | 100 | 1,614 | 100 |

Table. S8 Gene function annotation of predicted genes.

| Item | Hap A | | Hap B | |
| --- | --- | --- | --- | --- |
|  | Count | Percentage | Count | Percentage |
| All | 31,688 | 100.00% | 32,011 | 100.00% |
| Annotation | 29,388 | 92.74% | 29,708 | 92.81% |
| KEGG | 7,101 | 22.41% | 7,299 | 22.80% |
| Pathway | 5,606 | 17.69% | 5,740 | 17.93% |
| Nr | 27,633 | 87.20% | 27,862 | 87.04% |
| Uniprot | 27,251 | 86.00% | 27,472 | 85.82% |
| GO | 20,114 | 63.48% | 20,355 | 63.59% |
| Pfam | 798 | 2.52% | 751 | 2.35% |
| Interpro | 20,707 | 65.35% | 20,951 | 65.45% |

Table. S9 Summary of non-coding RNA annotation results

| Genome version | Type |  | Copy | Average length(bp) | | Total length(bp) | % of genome |
| --- | --- | --- | --- | --- | --- | --- | --- |
| Hap A | miRNA |  | 162 | | 122 | 19,832 | 0.004916 |
|  | tRNA |  | 534 | | 75 | 40,072 | 0.009934 |
|  | rRNA | rRNA | 333 | | 139 | 46,327 | 0.011484 |
|  |  | 18S | 4 | | 1567 | 6,268 | 0.001554 |
|  |  | 28S | 11 | | 198 | 2,174 | 0.000539 |
|  |  | 5.8S | 4 | | 156 | 624 | 0.000155 |
|  |  | 5S | 314 | | 119 | 37,261 | 0.009237 |
|  | snRNA | snRNA | 2,135 | | 110 | 234,496 | 0.05813 |
|  |  | CD-box | 1,961 | | 107 | 210,481 | 0.052177 |
|  |  | HACA-box | 39 | | 131 | 5,097 | 0.001264 |
|  |  | splicing | 135 | | 140 | 18,918 | 0.00469 |
|  |  | scaRNA | 0 | | 0 | 0 | 0 |
| Hap B | miRNA |  | 151 | | 122 | 18,411 | 0.004553 |
|  | tRNA |  | 533 | | 75 | 39,871 | 0.00986 |
|  | rRNA | rRNA | 267 | | 159 | 42,382 | 0.010481 |
|  |  | 18S | 9 | | 1143 | 10,290 | 0.002545 |
|  |  | 28S | 12 | | 197 | 2,369 | 0.000586 |
|  |  | 5.8S | 9 | | 166 | 1,494 | 0.000369 |
|  |  | 5S | 237 | | 119 | 28,229 | 0.006981 |
|  | snRNA | snRNA | 2,078 | | 110 | 228,516 | 0.056509 |
|  |  | CD-box | 1,902 | | 107 | 204,389 | 0.050543 |
|  |  | HACA-box | 41 | | 129 | 5,293 | 0.001309 |
|  |  | splicing | 135 | | 140 | 18,834 | 0.004657 |
|  |  | scaRNA | 0 | | 0 | 0 | 0 |

Table. S10 Summary of gene family cluster results

| Species | Genes number | Genes in families | Family number | Unique families | Average genes per family | |
| --- | --- | --- | --- | --- | --- | --- |
| *M.suaveolens.A* | 31,688 | 31,688 | 18,623 | 1,334 | | 1.70 |
| *M.suaveolens.B* | 32,011 | 32,011 | 18,774 | 1,412 | | 1.71 |
| *O.sativa* | 28,556 | 28,556 | 15,572 | 3,881 | | 1.83 |
| *S.baicalensis* | 28,315 | 28,315 | 17,063 | 3,203 | | 1.66 |
| *S.miltiorrhiza* | 26,467 | 26,467 | 15,377 | 1,262 | | 1.72 |
| *S.splendens* | 53,408 | 53,408 | 15,984 | 2,088 | | 3.34 |
| *T.quinquecostatus* | 28,964 | 28,964 | 15,486 | 925 | | 1.87 |
| *A.carvifolia* | 47,581 | 47,581 | 24,532 | 11,349 | | 1.94 |
| *V.vinifera* | 23,180 | 23,180 | 13,772 | 949 | | 1.68 |
| *A.thaliana* | 27,444 | 27,444 | 15,579 | 3,148 | | 1.76 |
| *L.esculentum* | 25,158 | 25,158 | 14,229 | 1,021 | | 1.77 |
| *S.indicum* | 22,661 | 22,661 | 13,865 | 535 | | 1.63 |
| *C.roseus* | 34,363 | 34,363 | 15,437 | 2,676 | | 2.23 |
| *S.tenuifolia* | 27,971 | 27,971 | 14,518 | 1,254 | | 1.93 |
| *M.longifolia* | 20,071 | 20,071 | 11,492 | 1,134 | | 1.75 |

Table. S11 Analysis of SNP and InDel mutations.

|  | **SNP** | **InDel** |
| --- | --- | --- |
| **Number** |  |  |
| Gene | 1,105,920 | 260,877 |
| Intergenic | 1,815,737 | 284,839 |
| **Structure** |  |  |
| UTR3 | 32,285 | 84,087 |
| UTR5 | 24,488 | 86,847 |
| Downstream | 157,522 | 352 |
| Upstream | 189,775 | 1,568 |
| Upstream/downstream | 21,879 | 0 |
| Intronic | 506,193 | 0 |
| Exonic | 172,852 | 0 |
| Intergenic | 1,815,737 | 0 |
| Splicing | 852 | 84,087 |
| **Function** |  |  |
| Synonymous SNV | 10,233 | 0 |
| Nonsynonymous_SNV | 8,636 | 0 |
| Stoploss | 46,029 | 49 |
| Stopgain | 60,189 | 208 |
| Nonframeshift_insertion | 8,125 | 1,924 |
| Nonframeshift_insertion | 117,437 | 1,938 |
| Frameshift_insertion | 9,915 | 2,733 |
| Frameshift_deletion | 284,839 | 3,063 |

Table. S12 With known functions genes which participates in menthol monoterpene biosynthesis in mint related species.

| Species | Gene ID | NCBI number | Function |
| --- | --- | --- | --- |
| *Salvia miltiorrhiza* | DXS | JN831118 | D-xylulose-5-phosphate synthase |
| *Salvia miltiorrhiza* | DXR | FJ476255 | D-xylulose-5-phosphate reductoisomerase |
| *Salvia miltiorrhiza* | MCT | AEZ55666 | 2-C-methyl-D-erythritol-4-phosphate cytidylyltransferase |
| *Plectranthus barbatus* | CMT | KU178947 | 4-diphosphocytidyl-2-C-methyl-D-erythritol Kinase |
| *Perilla frutescens var. hirtella* | MDS | KAH6830028 | 2-C-methyl-D-erythritol 2,4-cyclodiphosphate synthase |
| *Osmanthus fragrans* | HDS | KX400847 | 4-hydroxy-3-methylbut-2-en-1-yl diphosphate synthase |
| *Mentha x piperita* | HDR | KY888887 | 4-hydroxy-3-methylbut2-en-1-yl diphosphate reductase |
| *Mentha spicata* | LS | Q40322 | Limonene synthase |
| *Mentha x piperita* | L3H | Q9XHE7 | Limonene-3-hydroxylase |
| *Mentha x gracilis* | L6H | AY281025 | Limonene-6-hydroxylase |
| *Mentha x piperita* | ISPD | Q5C9I9 | Isopiperitenone dehydrogenase |
| *Mentha x piperita* | ISPR | Q6WAU1 | Isopiperitenone reductase |
| *Mentha x piperita* | MR | AY288137 | Menthol reductase |

Table. S13 Identification of gene families involved in monoterpene biosynthesis in *M. suaveolens*.

| ID | Gene name | Chr | Homology to canonical genes  (Amino acid level) |
| --- | --- | --- | --- |
| MsuaChr5AG00132300 | MsADXR | 5 | 94.31% identity to JN831118 |
| MsuaChr2AG00098990 | MsADXS | 2 | 82.62% identity to FJ476255 |
| MsuaChr11AG00266190 | MsAMCT | 11 | 90.13% identity to AEZ55666 |
| MsuaChr6AG00150920 | MsACMT | 6 | 85.82% identity to KU178947 |
| MsuaChr3AG00085090 | MsAMDS | 3 | 87.76% identity to KAH6830028 |
| MsuaChr4AG00039830 | MsAHDS | 4 | 89.08% identity to KX400847 |
| MsuaChr11AG00271940 | MsAHDR | 11 | 98.92% identity to KY888887 |
| MsuaChr5AG00133040 | MsALS1 | 5 | 96.83% identity to Q40322 |
| MsuaChr11AG00268330 | MsALS2 | 11 | 76.82% identity to Q40322 |
| MsuaChr11AG00268320 | MsALS3 | 11 | 74.62% identity to Q40322 |
| MsuaChr11AG00268340 | MsALS4 | 11 | 72.67% identity to Q40322 |
| MsuaChr5AG00133060 | MsAL3H1 | 5 | 98.2% identity to Q9XHE |
| MsuaChr9AG00283510 | MsAL3H2 | 9 | 67.32% identity to Q9XHE |
| MsuaChr9AG00279820 | MsAL3H3 | 9 | 60.12% identity to Q9XHE |
| MsuaChr2AG00097830 | MsAISPD1 | 2 | 98.87% identity to Q5C9I |
| MsuaChr2AG00097840 | MsAISPD2 | 2 | 72.79% identity to Q5C9I |
| MsuaChr11AG00255530 | MsAISPR1 | 11 | 79.18% identity to Q6WAU1 |
| MsuaChr11AG00255550 | MsAISPR2 | 11 | 93.22% identity to Q6WAU1 |
| MsuaChr11AG00255570 | MsAISPR3 | 11 | 62.15% identity to Q6WAU1 |
| MsuaChr11AG00255500 | MsAMR1 | 11 | 89.35% identity to AY288137 |
| MsuaChr11AG00255540 | MsAMR2 | 11 | 69.97% identity to AY288137 |
| MsuaChr11AG00255560 | MsAMR3 | 11 | 77.17% identity to AY288137 |

Table. S14 Identification of SVs affect genes

| **Item** | **Number** | **Percentage** |
| --- | --- | --- |
| The total number of gene | 31,688 |  |
| SVs affect genes | 5,493 | 17.3% |
| gene number in monoterpenoid pathway | 27 | 0.49% |
| SVs affect gene in monoterpenoid pathway | 11 | 0.20% |

Table. S15 NTF2 with known functions in other species of in NCBI.

| Species | Gene ID | NCBI number |
| --- | --- | --- |
| *Actinidia chinensis* | NTF2-L | PSS30325 |
| *Dichanthelium oligosanthes* | NTF2 | OEL12559 |
| *Panicum miliaceum* | NTF2-L | RLM86737 |
| *Oryza sativa* | NTF2 | Q9XJ54 |
| *Panicum miliaceum* | NTF2 | RLN03899 |
| *Zea mays* | NTF2 | NP_001131358 |

Table. S16 CYP71D with known functions in other species in NCBI.

| Species | Gene ID | NCBI number |
| --- | --- | --- |
| *Euphorbia peplus* | MW594405 | CYP71D365 |
| *Euphorbia lathyris* | KR350668 | CYP71D445 |
| *Euphorbia pekinensis* | MF034929 | CYP71D451 |
| *Morus notabilis* | XM_024169858 | CYP71D10 |
| *Ruta graveolens* | AY684276 | CYP71D |
| *Solanum habrochaites* | MT786523 | CYP71D184 |
| *Nicotiana tabacum* | KC747733 | CYP71D |
| *Nicotiana tabacum* | DQ350344 | CYP71D51v1 |
| *Nicotiana tabacum* | DQ350345 | CYP71D51v2 |
| *Nicotiana tabacum* | KC480444 | CYP71D51 |
| *Nicotiana tabacum* | DQ350346 | CYP71D51v3 |
